# DeepSense: An Adaptive Scalable Ensemble Framework for Industrial IoT Anomaly Detection

**DOI:** 10.3390/s26092662

**Published:** 2026-04-24

**Authors:** Amir Firouzi, Ali A. Ghorbani

**Affiliations:** Faculty of Computer Science, University of New Brunswick (UNB), Fredericton, NB E3B 5A3, Canada

**Keywords:** Industrial Internet of Things (IIoT), Internet of Things (IoT), IIoT security, intrusion detection system (IDS), anomaly detection, machine learning, deep learning, adaptive ensemble learning, cyber–physical systems security

## Abstract

The Industrial Internet of Things (IIoT) has become a cornerstone of modern industrial automation, enabling real-time monitoring, intelligent decision-making, and large-scale connectivity across cyber–physical systems. However, the growing scale, heterogeneity, and dynamic behavior of IIoT environments significantly expand the attack surface and challenge the effectiveness of conventional security mechanisms. In this paper, we propose DeepSense, a hybrid and adaptive anomaly and intrusion detection framework specifically designed for resource-constrained and heterogeneous IIoT deployments. DeepSense integrates three complementary components: DataSense, a realistic data pipeline and experimental testbed supporting synchronized sensor and network data processing; RuleSense, a lightweight rule-based detection layer that provides fast, deterministic, and interpretable anomaly screening at the edge; and NeuroSense, a learning-driven detection module comprising an adaptive ensemble of 22 machine learning and deep learning models spanning classical, neural, hybrid, and Transformer-based architectures. NeuroSense operates as a second detection stage that validates suspicious events flagged by RuleSense and enables both coarse-grained and fine-grained attack classification. To support rigorous and practical assessment, this work further introduces a comprehensive performance evaluation framework that extends beyond accuracy-centric metrics by jointly considering detection quality, latency, resource efficiency, and detection coverage, alongside an optimization-based process for selecting Pareto-optimal model ensembles under realistic IIoT constraints. Extensive experiments across diverse detection scenarios demonstrate that DeepSense exhibits strong generalization, lower false positive rates, and robust performance under evolving attack behaviors. The proposed framework provides a scalable and efficient IIoT security solution that meets the operational requirements of Industry 4.0 and the resilience-oriented objectives of Industry 5.0.

## 1. Introduction

The Industrial Internet of Things (IIoT) refers to the large-scale integration of networked sensors, actuators, controllers, and edge or cloud intelligence within industrial cyber–physical systems to enable the continuous monitoring, control, and data-driven optimization of physical processes [1]. In contrast to consumer IoT, IIoT deployments operate under stringent requirements for availability, safety, latency, and reliability, and typically span layered architectures that tightly couple sensing, communication, middleware, and application services [2]. This convergence has significantly accelerated the digitalization of industrial environments, while simultaneously increasing operational complexity and expanding the security attack surface of connected production systems [3,4].

Across modern Industry 4.0 ecosystems, IIoT enables pervasive data acquisition and distributed intelligence by combining sensing infrastructures with edge or cloud computing and machine learning, thereby supporting near-real-time decision-making under bandwidth and latency constraints [5]. Recent studies emphasize that the joint adoption of IoT, edge computing, cloud infrastructures, and AI has become a dominant engineering paradigm for scalable analytics and operational intelligence in connected industrial environments [4]. In parallel, IIoT increasingly underpins digital-twin-enabled manufacturing, where real-time data streams synchronize physical assets with virtual counterparts for prediction, optimization, and rapid what-if analysis [6].

This transformation is visible across a wide range of high-impact applications. In smart manufacturing, IIoT supports predictive maintenance, condition monitoring, and adaptive production workflows through continuous telemetry and AI-based inference, often mediated by digital twin models [5,6]. In healthcare, IoT-connected sensing and monitoring systems enable remote patient supervision and personalized services, while simultaneously introducing stringent requirements for the confidentiality, integrity, and interoperability of sensitive medical data flows [7]. In critical energy infrastructure, the transition toward digitally operated and interconnected smart-grid assets improves observability and automation, but it also increases the exposure of power systems to cyber threats that can propagate into physical disruptions and large-scale service outages [8].

However, the expanded attack surface introduced by IIoT connectivity and industrial control system integration makes security a first-order design constraint rather than a secondary consideration [3]. Surveyed evidence indicates that IIoT threats span multiple layers, including device compromise, protocol manipulation, lateral movement across OT and IT boundaries, and data integrity attacks targeting monitoring and control pipelines [9]. Historical analyses of ICS-focused cyber incidents show that adversaries increasingly target safety and operational continuity, and that successful compromises can result in cascading societal impacts such as prolonged service outages, equipment damage, and safety hazards [10]. More recent incident datasets focusing on the energy sector further demonstrate that cyber events affecting critical infrastructure remain persistent and continue to evolve in scale and sophistication, reinforcing the urgency of robust detection and response mechanisms [11].

Addressing IIoT security in practice remains challenging due to heterogeneous devices and protocols, imbalanced and evolving attack distributions, real-time operational constraints, limited labeled data, and the need for generalization across sites and operating regimes [9,12]. Although machine learning and deep learning intrusion detection systems (IDS) can outperform static rule-based defenses by learning complex traffic and behavioral patterns [13,14], recent surveys emphasize that many existing approaches still suffer from limited interpretability, dataset bias, deployment overhead, and reduced robustness under distribution shift and adaptive adversaries [12]. At the same time, modern attention-based models, including Transformer-based and hybrid architectures, are increasingly explored for IoT and IIoT intrusion detection due to their ability to model long-range dependencies and multi-scale patterns [15,16]. Nevertheless, open challenges remain regarding computational efficiency, explainability, and reliable operation in resource-constrained and safety-critical IIoT environments [17]. These limitations motivate the design of unified detection frameworks that combine complementary detection stages and multi-granularity classification to reduce false alarms while improving actionable attack attribution.

In this paper, we propose DeepSense, a hybrid anomaly and intrusion detection framework designed to address the security challenges inherent in large-scale and heterogeneous Industrial Internet of Things (IIoT) environments. DeepSense is motivated by the expanding attack surface introduced by pervasive connectivity, resource-constrained devices, and tight cyber–physical integration, which renders conventional security mechanisms inadequate in terms of adaptability, latency, and robustness. The proposed framework integrates a lightweight rule-based detection layer (RuleSense) for fast, interpretable, and low-overhead anomaly screening at the edge with an adaptive learning-driven layer (NeuroSense) that employs an ensemble of machine learning and deep learning models to capture complex spatial and temporal attack patterns and to enable both coarse-grained and fine-grained attack classification. This adaptive ensemble design improves generalization across heterogeneous IIoT deployments and enhances resilience against evolving and previously unseen attack strategies. In addition, DataSense provides a modular data pipeline supporting feature extraction, selection, profiling, and systematic evaluation, facilitating reproducibility and deployment across diverse industrial settings.

Beyond architectural contributions, this work also proposes a comprehensive performance evaluation framework that extends beyond conventional accuracy-centric metrics by jointly assessing detection quality, response speed and latency, resource efficiency, and detection coverage. By unifying fast edge-level response, adaptive ensemble intelligence, and multidimensional performance assessment, DeepSense delivers a scalable, efficient, and resilient security solution aligned with the real-time operational constraints of Industry 4.0 and the emerging resilience- and human-centric objectives of Industry 5.0.

The key contributions of this work are summarized as follows:

- Proposed a new multi-layer IIoT intrusion detection architecture that combines the previously evaluated standalone models into an adaptive ensemble layer jointly operating with a lightweight rule-based screening layer.
- Extended the previously introduced DataSense data infrastructure into a deployable framework component that supports online feature preparation, device profiling, and interaction with adaptive detection layers within the full DeepSense architecture.
- Proposed and empirically validated a comprehensive performance evaluation framework that extends beyond accuracy-centric metrics to jointly assess detection quality, response speed and latency, resource efficiency, and detection coverage.
- Proposed and implemented a practical window-based adaptive retraining, reprofiling, and drift monitoring mechanism to mitigate detection performance degradation caused by environmental dynamics and concept drift in long-running IIoT deployments.
- Proposed and implemented a lightweight and effective decision fusion mechanism within the DeepSense framework that aggregates outputs from heterogeneous detection methods, improving robustness and reducing false positives while maintaining suitability for resource-constrained IIoT environments.
- Proposed and implemented a theoretically grounded optimization process for selecting an effective ensemble of detection models within NeuroSense, maximizing detection performance while explicitly considering practical IIoT constraints such as computational overhead, memory usage, and inference latency.
- Performed extensive experimental evaluations across diverse operational scenarios and threat models, demonstrating the effectiveness, robustness, and practical trade-offs of the integrated DeepSense framework under competing performance and efficiency metrics.

The remainder of this paper is structured as follows. Section 2 reviews existing and related approaches for addressing anomaly and intrusion detection challenges in IoT and IIoT environments. Section 3 presents the architecture of the proposed DeepSense framework, detailing its core layers and components, including the adaptive ensemble learning strategy, the lightweight fusion engine, and the concept drift detection and adaptation mechanisms. Section 4 introduces the proposed performance evaluation framework, formally defining its theoretical characteristics and evaluation metrics, and describing the optimization-based process for selecting Pareto-optimal ensembles under realistic IIoT constraints. Section 5 reports the empirical evaluation of DeepSense and its individual components using the proposed assessment methodology, analyzing ensemble selection, adaptive learning behavior, and their impact on generalization and interoperability across diverse detection scenarios. Finally, Section 6 concludes the paper and outlines promising directions for future research.

## 2. Related Work

This section reviews existing research on anomaly and intrusion detection in IIoT environments, categorizing prior work based on detection paradigms, learning strategies, adaptability to concept drift, and deployment considerations in resource-constrained industrial settings.

### 2.1. Lightweight and Edge-Oriented Intrusion Detection

Several studies focus on designing lightweight IDS solutions suitable for deployment on edge or resource-constrained IIoT devices. Al Rawajbeh et al. propose a real-time adaptive IDS based on online ensemble learning with SHAP-based explainability, achieving low latency and high interpretability on edge hardware [17]. Ismail et al. conduct a comprehensive comparison of lightweight ML classifiers across multiple IIoT datasets, emphasizing computational efficiency, model size, and cross-dataset generalization for practical deployments [13]. Laiq et al. address DDoS detection in Edge-IIoT networks using classical ML ensembles and XGBoost, highlighting the feasibility of ensemble learning under edge constraints [18].

### 2.2. Machine Learning and Ensemble-Based IDS for IIoT

A large body of work explores classical machine learning and ensemble techniques for IIoT intrusion detection. Mohy-eddine et al. propose an ensemble-based IDS combining feature selection, outlier removal, and Random Forest classification to improve detection accuracy on imbalanced IIoT datasets [19]. A similar ensemble-driven design is presented in [20], where Isolation Forest and Pearson correlation are combined with Random Forests to reduce dimensionality and inference time. Eid et al. evaluate multiple ML classifiers on the WUSTL-IIoT-2021 dataset, demonstrating the strong performance of Random Forests for IIoT intrusion detection [21]. These works demonstrate the effectiveness of ensemble and feature-engineered ML approaches but largely rely on static models.

### 2.3. Deep Learning and Attention-Based Detection Models

Deep learning has been widely adopted to capture complex temporal and spatial patterns in IIoT traffic. Nandanwar and Katarya propose an adaptive CNN–GRU architecture for botnet detection in IIoT networks, achieving high accuracy across multi-class attack scenarios [22]. Alshehri et al. introduce a self-attention-based deep CNN to handle imbalanced data and repetitive traffic patterns, demonstrating improved discrimination of attack classes [23]. Saheed et al. present a GA-optimized attention-enhanced LSTM model with SHAP-based explainability for multivariate sensor data in critical IIoT infrastructures [24]. Gueriani et al. further extend deep sequential modeling by combining BiGRU, LSTM, and multi-head attention to achieve cross-domain robustness across medical and industrial IoT environments [25].

### 2.4. Adaptive Learning and Concept Drift Handling

Addressing non-stationarity and concept drift is a critical challenge in long-running IIoT systems. Lin et al. propose an ensemble learning framework with explicit drift detection for highly imbalanced IIoT data, relying on offline classifiers and staged retraining [26]. Raeiszadeh et al. introduce a real-time adaptive anomaly detection method that integrates drift-aware prediction models for multi-dimensional IIoT data streams [27]. Yang et al. propose ASTREAM, which combines sliding windows, PCA, and change detection to enable scalable anomaly detection over infinite IIoT data streams [28]. More recent work by Li et al. introduces an online adaptive ensemble learning scheme with causal feature selection and proactive drift detection for IIoT production processes [29]. Related adaptive stream analytics frameworks are also explored in [30], emphasizing automated feature selection and window-based ensemble learning for Industry 5.0 systems.

### 2.5. Surveys and Foundational Studies

Several surveys and foundational works provide comprehensive perspectives on IIoT intelligence and security. Latif et al. present a broad survey of deep learning techniques, architectures, and applications in IIoT, highlighting key challenges and open research directions [31]. Chen et al. discuss the role of machine learning in enhancing the cognitive capabilities of edge-enabled IIoT systems, emphasizing adaptive intelligence at the network edge [32]. Yan et al. propose an adaptive learning-rate-based neural anomaly detection framework to improve scalability and trustworthiness in large-scale IIoT deployments [33].

### 2.6. Discussion and Research Gaps

While existing approaches demonstrate strong detection performance, most focus on isolated detection paradigms, rely on static or single-stage models, or evaluate effectiveness primarily through accuracy-centric metrics. Limited attention is given to unified multi-layer detection architectures, adaptive ensemble optimization under resource constraints, and holistic performance evaluation considering latency, efficiency, and coverage. These limitations motivate the proposed DeepSense framework, which integrates rule-based and learning-driven detection, adaptive ensemble selection, concept drift handling, and multidimensional performance assessment tailored for realistic IIoT environments.

## 3. Proposed DeepSense Framework

This section presents **DeepSense**, a hybrid anomaly detection framework designed for Industrial Internet of Things (IIoT) environments. DeepSense integrates three core components: *RuleSense*, a lightweight rule-based detection module for fast edge-level anomaly detection; *NeuroSense*, an ensemble of machine learning and deep learning models for adaptive attack classification; and *DataSense*, a modular data pipeline and testbed supporting feature extraction, feature selection, profiling, and evaluation. Together, these components enable scalable, interpretable, and responsive anomaly detection across heterogeneous IIoT deployments. A detailed description of the physical testbed, implementation environment, device configuration, and attack generation process is available in [34].

### 3.1. Framework Architecture

**Figure 1:**
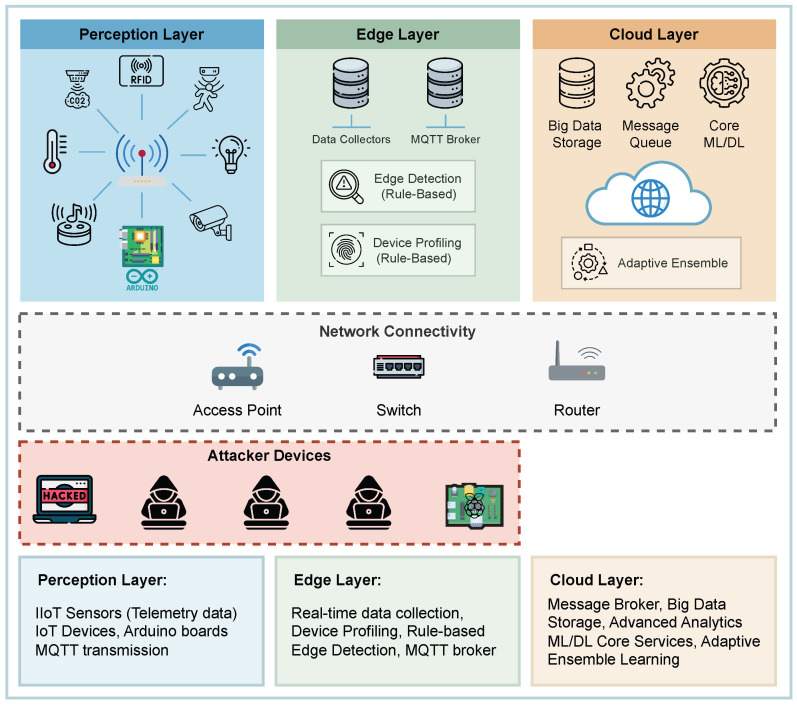
Framework architecture of the proposed DeepSense framework.

DeepSense follows a layered architecture comprising the *Perception*, *Edge*, and *Cloud* layers, as illustrated in Figure 1.

- **Perception Layer**: Interfaces with physical sensors and devices to collect telemetry data (e.g., temperature, motion, traffic flows). Data is transmitted via MQTT to higher layers.
- **Edge Layer**: Performs real-time processing and rule-based detection close to the data source. It constructs device behavior profiles and applies lightweight rules to flag anomalies, forwarding suspicious samples to the cloud for further analysis.
- **Cloud Layer**: Supports adaptive, scalable analysis using ensemble ML/DL models. It hosts rule generation, storage, and advanced classification components, facilitating long-term learning, detailed attribution, and threat intelligence.

This distributed design ensures fast local response while leveraging centralized intelligence for complex and evolving threats.

### 3.2. System Design

**Figure 2:**
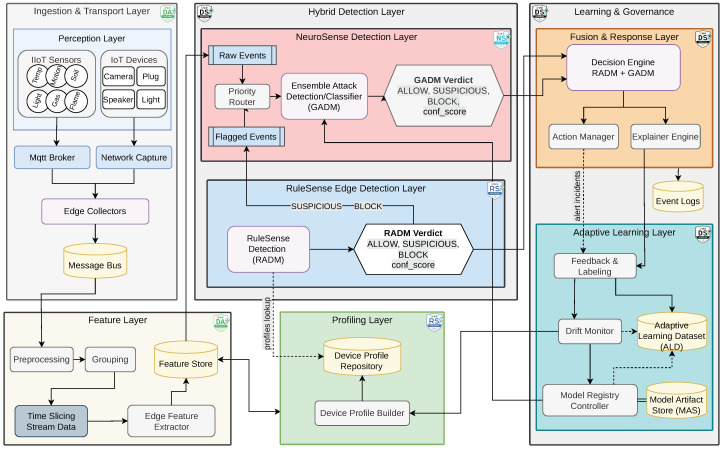
System design of the proposed DeepSense framework.

The internal structure of DeepSense is organized into seven functional layers, each contributing to end-to-end anomaly detection and system adaptability. These layers and their interactions are illustrated in Figure 2, which presents the detailed system design of the proposed framework. The major functional layers are summarized at a high level in this section, while detailed descriptions of their design and operational roles are provided in Appendix A.1, Appendix A.2 and Appendix A.3.

- **Ingestion & Transport Layer (DataSense)**: Aggregates heterogeneous IIoT data streams using MQTT and network capture modules. As the data acquisition backbone of DeepSense, this layer ensures reliable, low-latency delivery of synchronized sensor and network observations for downstream processing.
- **Feature Layer (DataSense)**: Preprocesses and transforms raw data into structured features using grouping, time slicing, and statistical extraction. Features are stored for inference and retraining.
- **Profiling Layer (RuleSense)**: Builds device behavior profiles from structured features. These profiles serve as baselines for real-time, rule-based anomaly detection at the edge.
- **Hybrid Detection Layer (DeepSense)**: Combines two submodules:
  - *RuleSense Edge Detection Layer*: Uses behavior profiles and detection rules to issue real-time anomaly verdicts (ALLOW, SUSPICIOUS, BLOCK), providing lightweight edge-level anomaly screening and early decision making.
  - *NeuroSense Detection Layer*: Applies an ensemble of ML/DL models to classify both flagged and raw samples, performing deeper validation, attack categorization, and fine-grained anomaly characterization with confidence scores.
- **Fusion & Response Layer (DeepSense)**: Fuses outputs from RuleSense and NeuroSense to generate unified verdicts. The decision engine enforces mitigation via an action manager, and provides interpretable outputs through an explainer engine.
- **Adaptive Learning Layer (DeepSense)**: Enables continuous improvement through feedback collection, drift detection, and dataset curation. It manages model retraining, versioning, and secure storage using a model registry and artifact store.

### 3.3. Adaptive Scalable Ensemble Implementation

**Figure 3:**
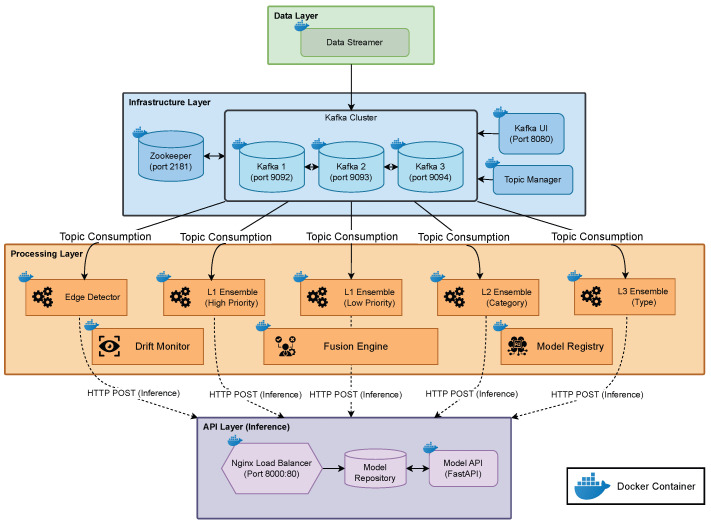
Architecture of the adaptive scalable ensemble framework for IIoT anomaly detection.

The DeepSense framework features an adaptive scalable ensemble architecture composed of four modular layers: the *Data Layer*, *Infrastructure Layer*, *Processing Layer*, and *API Layer*, as shown in Figure 3. This layered design supports high-throughput anomaly detection while ensuring adaptability in dynamic IIoT environments.

#### 3.3.1. Data and Infrastructure Layers

The Data Layer connects the framework to heterogeneous IIoT data streams, delivering sensor data into the pipeline without imposing processing overhead. The Infrastructure Layer supports asynchronous and resilient communication using a Kafka-based [35] messaging backbone coordinated by Zookeeper [36]. Kafka brokers handle topic-based data routing between processing modules, and custom topic managers aid in configurability and observability.

#### 3.3.2. Processing Layer and Detection Hierarchy

The Processing Layer is the core execution engine and it executes multi-stage anomaly detection. Incoming samples are first evaluated by *RuleSense*, a rule-based edge detector. Potential anomalies are then processed through a tiered ensemble: *L1* for binary detection (benign vs. attack), *L2* for attack category classification, and *L3* for attack type identification. At each layer, a fusion engine aggregates decisions from diverse base detectors.

#### 3.3.3. Model Registry and API Layer

The Model Registry orchestrates version-controlled retraining and deployment. The API Layer exposes RESTful endpoints (via FastAPI [37]) for all models, managed by an Nginx load balancer. This setup supports scalable inference and seamless integration across detection layers.

In summary, the adaptive scalable ensemble framework integrates modular streaming infrastructure, multi-layer detection, hierarchical decision fusion, and continuous drift monitoring to ensure robust and adaptable anomaly detection in dynamic IIoT environments. Although DeepSense includes multiple cooperating layers, each module is designed as an independent deployable component and can operate as a containerized service, allowing lightweight or full deployment depending on operational constraints, maintenance policies, and available computational resources. This modular structure also simplifies maintenance, since profiling logic, learning models, fusion policies, and retraining workflows can evolve independently without requiring full system redesign. Together, these design choices support real-time performance, progressive refinement, and automated adaptation, making the framework suitable for deployment in complex industrial settings with evolving threat landscapes.

#### 3.3.4. Fusion Engine

At each level, a dedicated **Fusion Engine** aggregates the outputs of *K* base detectors using confidence-weighted strategies. Let $Z_{t}$ represent the outcomes from *K* base detectors at time *t*, each providing a label $c_{i}$ and confidence score $s_{i}\in \left[0,1\right]$ with weight $w_{i}$.

For binary detection at Level 1, the fused attack probability $p_{t}$ is computed as:

(1) $$p_{t}=\frac{\sum_{i=1}^{K}w_{i}s_{i}}{\sum_{i=1}^{K}w_{i}},\;\hat{Y}t=I\left[p_{t}\geq \tau \right]$$

For L2/L3 multiclass decisions with *C* classes, weighted votes are aggregated as:

(2) $$v\left(c\right)=\sum_{i:c_{i}=c}w_{i},\;{\hat{p}}_{t}\left(c\right)=\frac{v(c)}{\sum_{c^{\prime }}v\left(c^{\prime }\right)},\;{\hat{Y}}_{t}=\arg\max_{c}{\hat{p}}_{t}\left(c\right)$$

These computations yield the final verdict ${\hat{Y}}_{t}$ and associated confidence $S_{t}=\max_{c}{\hat{p}}_{t}\left(c\right)$ (for multiclass) or $S_{t}=p_{t}$ (for binary).

#### 3.3.5. Concept Drift Detection

To ensure the continued reliability of anomaly detection under evolving data conditions, the DeepSense framework incorporates a two-stage concept drift detection mechanism. **Stage 1** continuously monitors detection performance using proper scoring rules, such as log loss and cross-entropy, over sliding windows to identify deviations in predictive reliability. **Stage 2** attributes detected drift to either increased false positives (Scenario A) or increased false negatives (Scenario B) by analyzing confusion-matrix statistics. This two-stage structure enables early and interpretable drift detection, while guiding appropriate adaptation actions based on the underlying cause.

The framework uses proper scoring rules to detect concept drift from prediction confidence and ground-truth labels. Drift is defined as a temporal change in the conditional distribution:

(3) $$P_{t}\left(Y|Z\right)\neq P_{t+\Delta t}\left(Y|Z\right)$$

##### Stage 1: Scoring-Based Detection

For L1 binary detection, the log loss is:

(4) $$l_{t}^{\left(L1\right)}=-\left[y_{t}\log p_{t}+\left(1-y_{t}\right)\log\left(1-p_{t}\right)\right]$$

with window-averaged score:

(5) $${\bar{l}}^{\left(L1\right)}\left(t\right)=\frac{1}{W}\sum_{i=t-W+1}^{t}l_{i}^{\left(L1\right)}$$

For L2/L3 multiclass detection, cross-entropy loss is used:

(6) $$l_{t}^{\left(L2/L3\right)}=-\log\left({\hat{p}}_{t}\left(Y_{t}\right)\right),\;{\bar{l}}^{\left(L2/L3\right)}\left(t\right)=\frac{1}{W}\sum_{i=t-W+1}^{t}l_{i}^{\left(L2/L3\right)}$$

##### Stage 2: Drift Attribution

Using confusion-matrix statistics over the window, drift is categorized:

(7) $$\begin{array}{cc} \mathrm{FPR}(t)=\frac{\mathrm{FP}(t)}{\mathrm{FP}(t)+\mathrm{TN}(t)}\; & \mathrm{FNR}(t)=\frac{\mathrm{FN}(t)}{\mathrm{FN}(t)+\mathrm{TP}(t)} \end{array}$$

A dominant rise in FPR indicates Scenario A (false alarms), while increased FNR indicates Scenario B (missed attacks).

##### Drift Response

Upon drift detection two different scenarios are considered in this work:

- Scenario A triggers rule re-profiling and fusion threshold calibration.
- Scenario B initiates model retraining via the Model Registry.

#### 3.3.6. Summary of the Drift Monitoring Procedure

The proposed concept drift detection mechanism is structured as a two-stage adaptive monitoring system. In Stage 1, the framework continuously evaluates layer-specific detection losses using proper scoring rules—log loss for binary detection (L1), and cross-entropy for multiclass classification (L2 and L3). These losses are monitored over a sliding window of *W* labeled samples. A drift alarm is triggered only if the windowed mean loss exceeds a statistically defined threshold for *M* consecutive windows, ensuring resilience against transient fluctuations and noise.

Once a drift is flagged, Stage 2 performs drift attribution to distinguish between two operational scenarios: Scenario A (increased false alarms due to evolving benign behavior) and Scenario B (increased misses due to adversarial changes in attack behavior). This is achieved by computing the false positive rate (FPR) and false negative rate (FNR) over the same detection window and applying a dominance rule. The outcome of this diagnosis directly guides the adaptation strategy: rule re-profiling and fusion calibration for Scenario A, and model retraining for Scenario B.

The overall procedure is formally presented in Algorithm 1.
**Algorithm 1:** Two-Stage Drift Detection and Attribution (Loss → FPR/FNR)
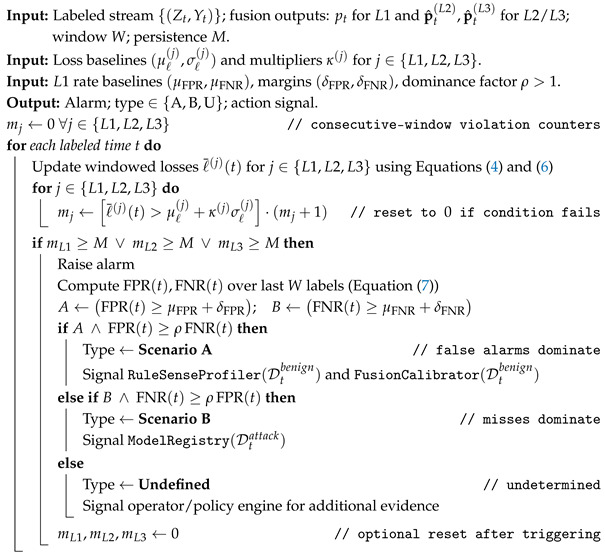


##### Windowed Adaptation Datasets

Once a drift alarm is raised at time *t*, the corresponding adaptation modules operate on the most recent window of labeled data. Two disjoint subsets are constructed based on the true labels:

(8) $$\begin{array}{cc} D_{t}^{\mathrm{benign}} & =\left\{\left(Z_{i},Y_{i}\right)\;:\;i\in \left[t-W+1,t\right],\;Y_{i}=0\right\},\\ D_{t}^{\mathrm{attack}} & =\left\{\left(Z_{i},Y_{i}\right)\;:\;i\in \left[t-W+1,t\right],\;Y_{i}=1\right\}. \end{array}$$

These subsets are passed to the appropriate adaptation module depending on the drift type. In Scenario A, $D_{t}^{\mathrm{benign}}$ is used for re-profiling the rule-based engine and recalibrating the fusion layer. In Scenario B, $D_{t}^{\mathrm{attack}}$ is utilized to retrain relevant models and update feature profiles via the Model Registry.

## 4. Performance Evaluation Framework

**Figure 4:**
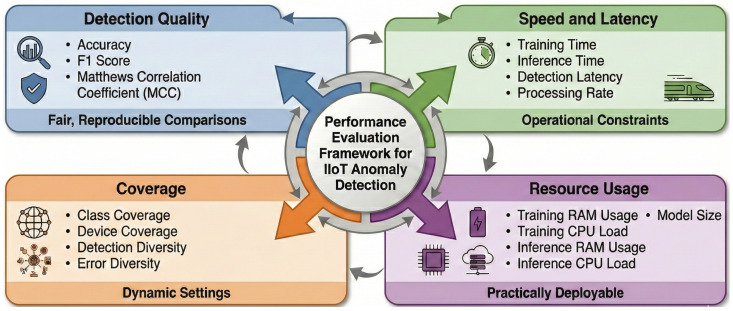
Proposed comprehensive Performance Evaluation Framework for IIoT anomaly detection.

The evaluation of anomaly detection models in Industrial Internet of Things (IIoT) environments must go beyond traditional accuracy metrics. Given the operational constraints of IIoT such as real-time response requirements, limited computational resources, and deployment heterogeneity, performance must be assessed along multiple axes. This section introduces a comprehensive and structured Performance Evaluation Framework that rigorously benchmarks detection approaches across four key dimensions: Detection Quality, Speed and Latency, Coverage, and Resource Usage. The overall design of the framework is illustrated in Figure 4, which outlines the evaluation dimensions, metric aggregation pipeline, and optimization-based ensemble selection. The framework is designed to support fair, reproducible comparisons and to guide the development of detection systems that are both robust and practically deployable in dynamic IIoT environments.

### 4.1. Overview and Motivation

Traditional evaluation metrics such as precision, recall, and accuracy, while useful, are insufficient for characterizing the real-world performance of anomaly detection systems deployed in IIoT environments. These systems must often operate on edge devices or within time-critical pipelines where decisions must be made quickly and efficiently. Moreover, IIoT networks face a wide variety of evolving threats, which demand that detection solutions maintain coverage across a broad and dynamic threat landscape.

### 4.2. Evaluation Dimensions and Metrics

To address these challenges, the proposed evaluation framework introduces a multidimensional approach that captures both algorithmic and operational aspects of detection performance. Specifically, it defines four core evaluation dimensions: (1) Detection Quality, which measures correctness and robustness of predictions; (2) Speed and Latency, reflecting real-time responsiveness; (3) Coverage, which evaluates the range of detectable attack types; and (4) Resource Usage, capturing computational and memory efficiency.

By adopting this structured approach, the framework ensures that detection methods are not only statistically effective but also viable for deployment in constrained and dynamic IIoT environments.

### 4.3. Detection Quality Metrics

Detection quality measures how reliably an anomaly detection model distinguishes benign from malicious behavior, which is critical in IIoT environments where both false alarms and missed attacks carry high operational cost. This dimension is evaluated using standard classification metrics derived from the confusion matrix of a model M, defined by true positives (TP), true negatives (TN), false positives (FP), and false negatives (FN), with total number of evaluation instances N=TP+TN+FP+FN.

**Accuracy** measures the overall proportion of correctly classified samples:

(9) $$\mathrm{Accuracy}\left(M\right)=\frac{TP+TN}{N}$$

While intuitive, accuracy can be misleading under class imbalance, which is common in IIoT traffic.

**F1 Score** provides a balanced measure of precision and recall, making it more suitable for imbalanced datasets:

(10) $$F1\left(M\right)=\frac{2\cdot TP}{2\cdot TP+FP+FN}$$

where

(11) $$\mathrm{Precision}=\frac{TP}{TP+FP}\;\mathrm{Recall}=\frac{TP}{TP+FN}.$$

If TP=0, the F1 score is defined as zero.

**Matthews Correlation Coefficient (MCC)** evaluates the correlation between predicted and true labels and remains informative even under severe class imbalance:

(12) $$\mathrm{MCC}\left(M\right)=\frac{TP\cdot TN-FP\cdot FN}{\sqrt{\left(TP+FP)(TP+FN)(TN+FP)(TN+FN\right)}}$$

MCC ranges from −1 (total disagreement) to +1 (perfect prediction), with 0 indicating random performance. If the denominator is zero, MCC is set to zero by convention.

These metrics jointly capture detection correctness and robustness, forming the basis for the reliable evaluation of IIoT anomaly detection models.

### 4.4. Speed and Latency Metrics

Real-time responsiveness is essential for anomaly detection in IIoT systems. This metric category evaluates a model M along four temporal aspects: training time, inference delay, detection latency, and throughput. Each metric is normalized to the range [0,1] for fair comparison, with higher values indicating better performance.

**Training Time** $T_{\mathrm{train}}\left(M\right)$ measures the elapsed time (in seconds) from model initialization to readiness, including preprocessing and profiling:

(13) $$T_{\mathrm{train}}\left(M\right)=\mathrm{Elapsed}\;\mathrm{time}\;\mathrm{for}\;\mathrm{preparing}\;\mathrm{model}\;M$$

Let $T_{\min},T_{\max}$ be the min and max training times across all models. The normalized score is:

(14) $${\tilde{T}}_{\mathrm{train}}\left(M\right)=1-\frac{T_{\mathrm{train}}\left(M\right)-T_{\min}}{T_{\max}-T_{\min}}$$

**Inference Time** per sample is computed as the average delay across *n* test samples, where $t_{i}$ is the inference time for the *i*-th sample and *T* is total elapsed inference time:

(15) $$T_{\mathrm{infer}}\left(M\right)=\frac{1}{n}\sum_{i=1}^{n}t_{i}\;\mathrm{or}\;T_{\mathrm{infer}}\left(M\right)=\frac{T}{n}$$

Normalized inference score using observed min and max inference times:

(16) $${\tilde{T}}_{\mathrm{infer}}\left(M\right)=1-\frac{T_{\mathrm{infer}}\left(M\right)-T_{\min}}{T_{\max}-T_{\min}}$$

**Detection Latency** L(M) is defined over *k* correctly detected attack events, where $\tau_{i}$ is the onset time and ${\hat{\tau }}_{i}$ is the detection time of the *i*-th attack:

(17) $$L\left(M\right)=\frac{1}{k}\sum_{i=1}^{k}\left({\hat{\tau }}_{i}-\tau_{i}\right)$$

Let $L_{\min},L_{\max}$ be the min and max latencies across models. The normalized latency score is:

(18) $$\tilde{L}\left(M\right)=1-\frac{L\left(M\right)-L_{\min}}{L_{\max}-L_{\min}}$$

**Processing Rate** R(M) (samples/second) is the throughput over inference time:

(19) $$R\left(M\right)=\frac{n}{T}$$

Let $R_{\min},R_{\max}$ denote the min and max rates across models. The normalized score is:

(20) $$\tilde{R}\left(M\right)=\frac{R\left(M\right)-R_{\min}}{R_{\max}-R_{\min}}$$

Together, these metrics quantify the temporal efficiency and operational readiness of a detection model under realistic IIoT constraints.

### 4.5. Coverage Metrics

Coverage metrics assess a model’s ability to generalize across diverse attack classes, device types, and detection scenarios in IIoT environments. Let $A=\{a_{1},\ldots ,a_{K}\}$ denote the set of attack classes, $D=\{d_{1},\ldots ,d_{M}\}$ the set of device types, *S* the set of all evaluation samples, and M the model under evaluation.

**Class Coverage** measures per-class detection accuracy, weighted by class importance $w_{k}$, where $\sum_{k=1}^{K}w_{k}=1$. For each attack class $a_{k}$, let $S_{a_{k}}\subseteq S$ denote the set of evaluation samples belonging to class $a_{k}$, and let $C_{M}\left(a_{k}\right)\subseteq S_{a_{k}}$ denote the subset of those samples correctly detected by model M:

(21) $$\mathrm{ClassCoverage}\left(M\right)=\sum_{k=1}^{K}w_{k}\cdot \frac{|C_{M}\left(a_{k}\right)|}{|S_{a_{k}}|}$$

The normalized score is:

(22) $$\tilde{\mathrm{ClassCoverage}}\left(M\right)=\frac{\mathrm{ClassCoverage}\left(M\right)-C_{\min}}{C_{\max}-C_{\min}}$$

**Device Coverage** measures detection consistency across devices, weighted by device importance $v_{m}$, where $\sum_{m=1}^{M}v_{m}=1$. For each device $d_{m}$, let $S_{d_{m}}\subseteq S$ be the set of samples originating from device $d_{m}$, and let $C_{M}\left(d_{m}\right)\subseteq S_{d_{m}}$ denote the subset of those correctly detected by model M:

(23) $$\mathrm{DeviceCoverage}\left(M\right)=\sum_{m=1}^{M}v_{m}\cdot \frac{|C_{M}\left(d_{m}\right)|}{|S_{d_{m}}|}$$

The normalized score is:

(24) $$\tilde{\mathrm{DeviceCoverage}}\left(M\right)=\frac{\mathrm{DeviceCoverage}\left(M\right)-D_{\min}}{D_{\max}-D_{\min}}$$

**Detection Diversity** quantifies the proportion of correct detections made exclusively by model M. Let $C_{M}\subseteq S$ be the set of samples correctly detected by M, and let $C_{\mathrm{others}}={⋃}_{M^{\prime }\neq M}C_{M^{\prime }}$ be the union of correct detections from all other models. The diversity score is defined as:

(25) $$\mathrm{DetectionDiversity}\left(M\right)=1-\frac{|C_{M}\cap C_{\mathrm{others}}|}{|C_{M}\cup C_{\mathrm{others}}|}$$

**Error Diversity** measures how distinct the misclassifications of model M are compared to those of other models. Let $E_{M}\subseteq S$ be the set of samples misclassified by M, and let $E_{\mathrm{others}}={⋃}_{M^{\prime }\neq M}E_{M^{\prime }}$ represent the union of misclassified samples from all other models. The subset of errors unique to M is:

(26) $$U_{M}^{\mathrm{error}}=E_{M}\setminus E_{\mathrm{others}}$$

Then the Error Diversity score is given by:

(27) $$\mathrm{ErrorDiversity}\left(M\right)=\frac{\left|U_{M}^{\mathrm{error}}\right|}{\left|E_{M}\right|}$$

These metrics collectively reflect a model’s robustness and its value in ensemble or deployment across varied IIoT conditions.

### 4.6. Resource Usage Metrics

IIoT environments demand lightweight detection solutions due to strict resource constraints. This category evaluates the computational footprint of a model M during training and inference, focusing on RAM, CPU, and storage usage.

Let:

- $m_{i}$: RAM usage (in MB) at training time step *i*; $m_{i}^{\left(\mathrm{eval}\right)}$: during inference.
- $c_{i}$: CPU usage (fraction in [0, 1]) at training time step *i*; $c_{i}^{\left(\mathrm{eval}\right)}$: during inference.
- *n*: Number of monitoring intervals.
- $M_{\min},M_{\max}$: Min/max RAM usage across models; similarly for CPU: $C_{\min},C_{\max}$, and model size: $S_{\min},S_{\max}$.

**Training RAM Usage** This metric reflects the peak memory required during training. It uses the sampled memory values $m_{i}$ to compute:

(28) $${\mathrm{MEM}}_{\mathrm{train}}\left(M\right)=\max_{1\leq i\leq n}m_{i},\;{\tilde{\mathrm{MEM}}}_{\mathrm{train}}\left(M\right)=1-\frac{{\mathrm{MEM}}_{\mathrm{train}}\left(M\right)-M_{\min}}{M_{\max}-M_{\min}}$$

**Training CPU Load** This metric measures the average processor usage over the training interval, computed from CPU samples $c_{i}$:

(29) $${\mathrm{CPU}}_{\mathrm{train}}\left(M\right)=\frac{1}{n}\sum_{i=1}^{n}c_{i},\;{\tilde{\mathrm{CPU}}}_{\mathrm{train}}\left(M\right)=1-\frac{{\mathrm{CPU}}_{\mathrm{train}}\left(M\right)-C_{\min}}{C_{\max}-C_{\min}}$$

**Inference RAM Usage** This metric reports the peak memory consumption during inference, based on $m_{i}^{\left(\mathrm{eval}\right)}$:

(30) $${\mathrm{MEM}}_{\mathrm{infer}}\left(M\right)=\max_{1\leq i\leq n}m_{i}^{\left(\mathrm{eval}\right)},\;{\tilde{\mathrm{MEM}}}_{\mathrm{infer}}\left(M\right)=1-\frac{{\mathrm{MEM}}_{\mathrm{infer}}\left(M\right)-M_{\min}^{\left(\mathrm{eval}\right)}}{M_{\max}^{\left(\mathrm{eval}\right)}-M_{\min}^{\left(\mathrm{eval}\right)}}$$

**Inference CPU Load** This metric captures the average processor usage during inference using $c_{i}^{\left(\mathrm{eval}\right)}$:

(31) $${\mathrm{CPU}}_{\mathrm{infer}}\left(M\right)=\frac{1}{n}\sum_{i=1}^{n}c_{i}^{\left(\mathrm{eval}\right)},\;{\tilde{\mathrm{CPU}}}_{\mathrm{infer}}\left(M\right)=1-\frac{{\mathrm{CPU}}_{\mathrm{infer}}\left(M\right)-C_{\min}^{\left(\mathrm{eval}\right)}}{C_{\max}^{\left(\mathrm{eval}\right)}-C_{\min}^{\left(\mathrm{eval}\right)}}$$

**Model Size** This metric reports the storage footprint of the model file in megabytes:

(32) $$\mathrm{ModelSize}\left(M\right)=\mathrm{Size}\;\mathrm{in}\;\mathrm{MB},\;\tilde{\mathrm{ModelSize}}\left(M\right)=1-\frac{\mathrm{ModelSize}\left(M\right)-S_{\min}}{S_{\max}-S_{\min}}$$

### 4.7. Metric Aggregation Techniques

In Industrial Internet of Things (IIoT) anomaly detection, model evaluation typically involves multiple performance metrics that reflect different and often conflicting objectives, such as detection quality, computational efficiency, response latency, and operational coverage. While individual metrics provide localized insights, they are insufficient for holistic model comparison in complex deployment scenarios. Relying on unaggregated metrics can lead to biased conclusions and obscure important trade-offs.

To address this limitation, we adopt a structured multi-metric aggregation strategy that maps sets of normalized performance metrics into interpretable scalar scores. Each evaluation dimension is treated as a multi-objective decision space, and aggregation is performed using distance-to-ideal techniques. This enables consistent comparison across models and supports downstream tasks such as ranking, selection, and multi-objective optimization.

#### 4.7.1. Dimension-Level Scoring

Let $M=\{M_{1},M_{2},\ldots ,M_{N}\}$ denote the set of evaluated models, and let $C=\{C_{1},C_{2},C_{3},C_{4}\}$ represent the core evaluation dimensions: Detection Quality, Resource Usage, Speed and Latency, and Coverage. Each dimension $C_{k}$ consists of $M_{k}$ metrics $\left\{m_{1}^{\left(k\right)},m_{2}^{\left(k\right)},\ldots ,m_{M_{k}}^{\left(k\right)}\right\}$, with associated weights $w^{\left(k\right)}={\left[w_{1}^{\left(k\right)},\ldots ,w_{M_{k}}^{\left(k\right)}\right]}^{⊤}$, where $\sum_{j=1}^{M_{k}}w_{j}^{\left(k\right)}=1$.

For a given dimension $C_{k}$, the performance of all models is organized into a decision matrix:

(33) $$X^{\left(k\right)}=\left[\begin{array}{cccc} x_{11}^{\left(k\right)} & x_{12}^{\left(k\right)} & \ldots & x_{1M_{k}}^{\left(k\right)}\\ x_{21}^{\left(k\right)} & x_{22}^{\left(k\right)} & \ldots & x_{2M_{k}}^{\left(k\right)}\\ \vdots & \vdots & \ddots & \vdots \\ x_{N1}^{\left(k\right)} & x_{N2}^{\left(k\right)} & \ldots & x_{NM_{k}}^{\left(k\right)} \end{array}\right],$$

where $x_{ij}^{\left(k\right)}$ denotes the raw performance value of model $M_{i}$ with respect to metric $m_{j}^{\left(k\right)}$.

Each metric is categorized as either a benefit criterion, where higher values are preferred, or a cost criterion, where lower values are preferred. To ensure comparability, all metrics are transformed to a common benefit-oriented scale in the interval [0,1] using min–max normalization:

(34) $$x_{ij}^{(k)\prime }=\left\{\begin{array}{cc} \frac{x_{ij}^{\left(k\right)}-\min_{i}x_{ij}^{\left(k\right)}}{\max_{i}x_{ij}^{\left(k\right)}-\min_{i}x_{ij}^{\left(k\right)}}, & \mathrm{if}\;m_{j}^{\left(k\right)}\;\mathrm{is}\;a\;\mathrm{benefit},\\ \frac{\max_{i}x_{ij}^{\left(k\right)}-x_{ij}^{\left(k\right)}}{\max_{i}x_{ij}^{\left(k\right)}-\min_{i}x_{ij}^{\left(k\right)}}, & \mathrm{if}\;m_{j}^{\left(k\right)}\;\mathrm{is}\;a\;\cos t. \end{array}\right.$$

The normalized decision matrix $X^{(k)\prime }$ is then weighted to obtain:

(35) $$V^{\left(k\right)}=X^{(k)\prime }\cdot w^{\left(k\right)}.$$

##### TOPSIS-Based Scoring

In the TOPSIS method, the ideal and anti-ideal solutions are defined as:

(36) $$v^{+}=\max_{i}v_{i}^{\left(k\right)},\;v^{-}=\min_{i}v_{i}^{\left(k\right)}.$$

The Euclidean distances of each model to these reference points are computed as:

(37) $$d_{i}^{+}={∥v_{i}^{\left(k\right)}-v^{+}∥}_{2},\;d_{i}^{-}={∥v_{i}^{\left(k\right)}-v^{-}∥}_{2}.$$

The resulting TOPSIS score is given by:

(38) $${\mathrm{Score}}_{\mathrm{TOPSIS}}^{\left(k\right)}\left(M_{i}\right)=\frac{d_{i}^{-}}{d_{i}^{+}+d_{i}^{-}}.$$

##### VIKOR-Based Scoring

VIKOR computes the group utility and individual regret for each model:

(39) $$\begin{array}{c} S_{i}=\sum_{j=1}^{M_{k}}w_{j}^{\left(k\right)}\cdot \frac{f_{j}^{*}-x_{ij}^{(k)\prime }}{f_{j}^{*}-f_{j}^{-}}, \end{array}$$

(40) $$\begin{array}{c} R_{i}=\max_{j}\left(w_{j}^{\left(k\right)}\cdot \frac{f_{j}^{*}-x_{ij}^{(k)\prime }}{f_{j}^{*}-f_{j}^{-}}\right), \end{array}$$

where $f_{j}^{*}=\max_{i}x_{ij}^{(k)\prime }$ and $f_{j}^{-}=\min_{i}x_{ij}^{(k)\prime }$.

The final VIKOR score is computed as:

(41) $${\mathrm{Score}}_{\mathrm{VIKOR}}^{\left(k\right)}\left(M_{i}\right)=1-\left(v\cdot \frac{S_{i}-S^{*}}{S^{-}-S^{*}}+\left(1-v\right)\cdot \frac{R_{i}-R^{*}}{R^{-}-R^{*}}\right),$$

where v∈[0,1] controls the trade-off between group utility and individual regret.

##### Augmented Chebyshev Scoring

To emphasize worst-case robustness, the Augmented Chebyshev method evaluates the maximum weighted deficit across metrics. The deficit matrix is defined as:

(42) $$\Delta =1-X^{(k)\prime }.$$

For each model $M_{i}$, the worst-case and cumulative deficits are computed as:

(43) $$\begin{array}{cc} d_{\infty }^{\left(i\right)} & =\max_{j}\left(w_{j}^{\left(k\right)}\cdot \Delta_{ij}\right), \end{array}$$

(44) $$\begin{array}{cc} d_{\mathrm{sum}}^{\left(i\right)} & =\sum_{j=1}^{M_{k}}w_{j}^{\left(k\right)}\cdot \Delta_{ij}. \end{array}$$

The augmented Chebyshev score is then defined as:

(45) $${\mathrm{Score}}_{\mathrm{AugChebyshev}}^{\left(k\right)}\left(M_{i}\right)=1-\left(d_{\infty }^{\left(i\right)}+\rho \cdot d_{\mathrm{sum}}^{\left(i\right)}\right),$$

where ρ∈(0,1) is a small augmentation coefficient.

This formulation prioritizes models with strong worst-case performance while maintaining sensitivity to cumulative deficiencies across metrics.

#### 4.7.2. Final Aggregation Vector and Method Comparison

For each model $M_{i}$, the result of dimension-level aggregation is a four-dimensional performance vector:

(46) $$A\left(M_{i}\right)=\left[{\mathrm{Score}}^{\left(1\right)}\left(M_{i}\right),\;{\mathrm{Score}}^{\left(2\right)}\left(M_{i}\right),\;{\mathrm{Score}}^{\left(3\right)}\left(M_{i}\right),\;{\mathrm{Score}}^{\left(4\right)}\left(M_{i}\right)\right],$$

where each score corresponds to a core evaluation dimension. This vector enables interpretable model comparison and serves as input to ranking or optimization procedures.

Different aggregation methods encode distinct decision preferences: TOPSIS favors overall balance by allowing compensation across metrics; VIKOR introduces compromise between average performance and worst-case regret; and Augmented Chebyshev emphasizes robustness by penalizing the weakest metric. The choice of method depends on the operational priorities of the target IIoT deployment.

### 4.8. Optimization-Based Ensemble Selection

After obtaining the aggregated performance vectors for each detection model across the four core evaluation dimensions including Detection Quality, Speed and Latency, Coverage, and Resource Usage, the final step in the evaluation framework involves selecting the most effective model combinations to form an ensemble. This is framed as a multi-objective subset selection problem, where the goal is to identify ensembles that simultaneously optimize all evaluation objectives.

We employ the Non-dominated Sorting Genetic Algorithm II (NSGA-II), a widely used multi-objective evolutionary algorithm, to explore the space of model subsets. Each candidate solution represents a binary selection vector indicating which models are included in the ensemble. The fitness of each candidate is evaluated using top-*k* average performance per dimension, favoring ensembles with multiple strong contributors across all objectives.

This optimization is applied independently to each hierarchical classification level: Level 1 (Binary), Level 2 (Attack Category), and Level 3 (Attack Type), reflecting different granularities of detection.

#### 4.8.1. Problem Formulation

Let $M_{1},\ldots ,M_{N}$ denote the set of detection models, and let each model $M_{i}$ have an aggregated score vector:

(47) $$A\left(M_{i}\right)=\left[{\mathrm{DQ}}_{i},\;{\mathrm{SL}}_{i},\;{\mathrm{COV}}_{i},\;{\mathrm{RU}}_{i}\right]\in {\left[0,1\right]}^{4}$$

The task is to identify subsets of models (ensembles) such that the combined performance lies on the Pareto front with respect to these four objectives. Each candidate ensemble is encoded as a binary vector $z\in {\left\{0,1\right\}}^{N}$, where $z_{i}=1$ indicates the inclusion of model $M_{i}$.

#### 4.8.2. NSGA-II Framework

For a candidate ensemble z, we define the fitness for each objective k∈{1,2,3,4} as the average performance among the top-*k* scoring models in the ensemble:

(48) $$f_{k}\left(z\right)=\frac{1}{|S_{k}|}\sum_{i\in S_{k}}A_{k}\left(M_{i}\right)$$

where:

- $S_{k}\subseteq \left\{i:z_{i}=1\right\}$ is the set of indices of the top models selected for objective *k*,
- $A_{k}\left(M_{i}\right)$ is the *k*-th dimension score for model $M_{i}$.

The full fitness vector for the ensemble is $f\left(z\right)=\left[f_{1},f_{2},f_{3},f_{4}\right]$. NSGA-II evolves a population of such candidates using binary tournament selection, crossover, and a mutation operator that adds, removes, or swaps models, constrained by a maximum ensemble size $K_{\max}$. The algorithm produces a Pareto front of non-dominated ensembles representing trade-off-optimal configurations.

#### 4.8.3. Post-Ranking with TOPSIS

To prioritize ensembles from the Pareto front, we apply a secondary ranking step using the TOPSIS method. Each ensemble’s objective vector is normalized within the Pareto front, and its proximity to the ideal point [1,1,1,1] and the anti-ideal point is calculated. The TOPSIS score is defined as:

(49) $${\mathrm{TOPSIS}}_{\mathrm{ensemble}}=\frac{d_{\mathrm{neg}}}{d_{\mathrm{pos}}+d_{\mathrm{neg}}}$$

where $d_{\mathrm{pos}}$ and $d_{\mathrm{neg}}$ are the Euclidean distances to the ideal and anti-ideal points, respectively. This post-ranking helps identify the most balanced ensembles for deployment.

#### 4.8.4. Output

The output of the optimization process for each classification level includes:

- The complete Pareto front of candidate ensembles
- A ranked list of ensembles based on TOPSIS scores
- The top-*k* ensembles recommended for deployment or further analysis

This section presented an optimization-based strategy for ensemble selection using NSGA-II. By modeling the selection task as a multi-objective problem over four normalized performance dimensions, we identified diverse, high-performing ensembles that represent optimal trade-offs. A final TOPSIS-based ranking stage facilitated selection of the most balanced configurations, supporting practical deployment across hierarchical classification levels in IIoT anomaly detection.

## 5. Experimental Results and Analysis

This section presents the empirical evaluation of the proposed *DeepSense* framework using the performance assessment methodology introduced in Section 4. The evaluation is structured around the unified multi-criteria performance framework, which allows a holistic comparison of heterogeneous detection and classification models under IIoT constraints. Experimental data are derived from our previously developed physical IIoT testbed [34], which includes heterogeneous devices, real communication protocols, and controlled attack scenarios. The results are organized into two main parts: (i) aggregated performance profiles of individual detection modules and learning-based classifiers, and (ii) the outcomes of the optimization-based ensemble selection process. For each component, performance metrics such as detection quality, resource usage, speed and latency, and coverage are reported and analyzed using vector-based scoring and Pareto optimization techniques. Visual summaries and tabular breakdowns are included to highlight trade-offs and identify configurations suitable for deployment at different layers of the DeepSense architecture. A detailed description of the physical testbed, implementation environment, and attack generation process is available in [34].

### 5.1. Aggregated Performance Metrics Across Models

**Figure 5:**
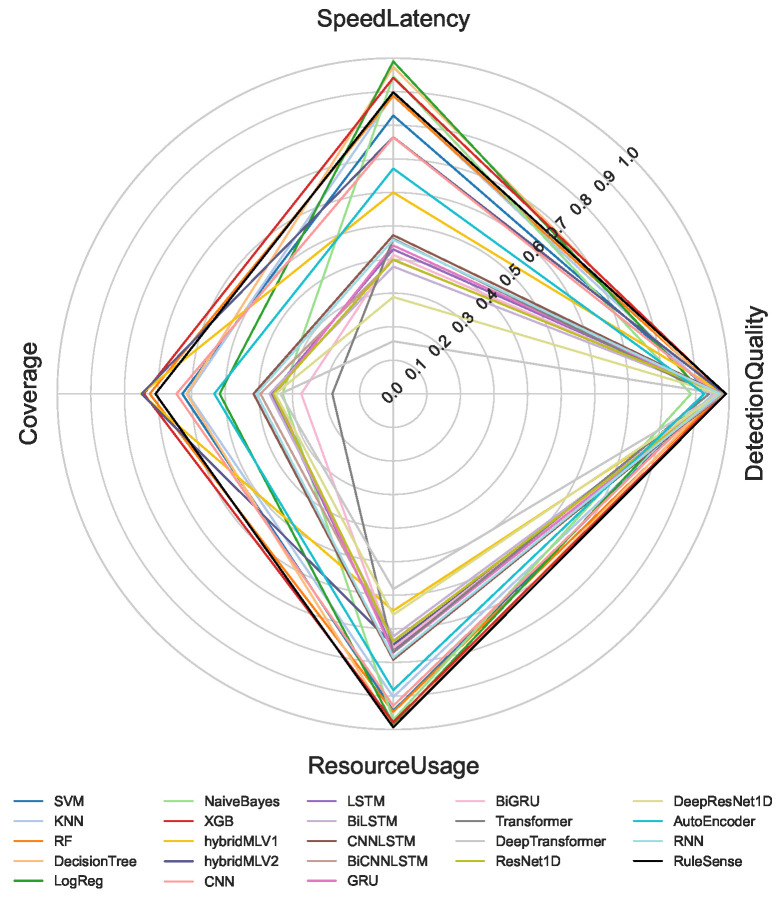
Binary Classification.

**Figure 6:**
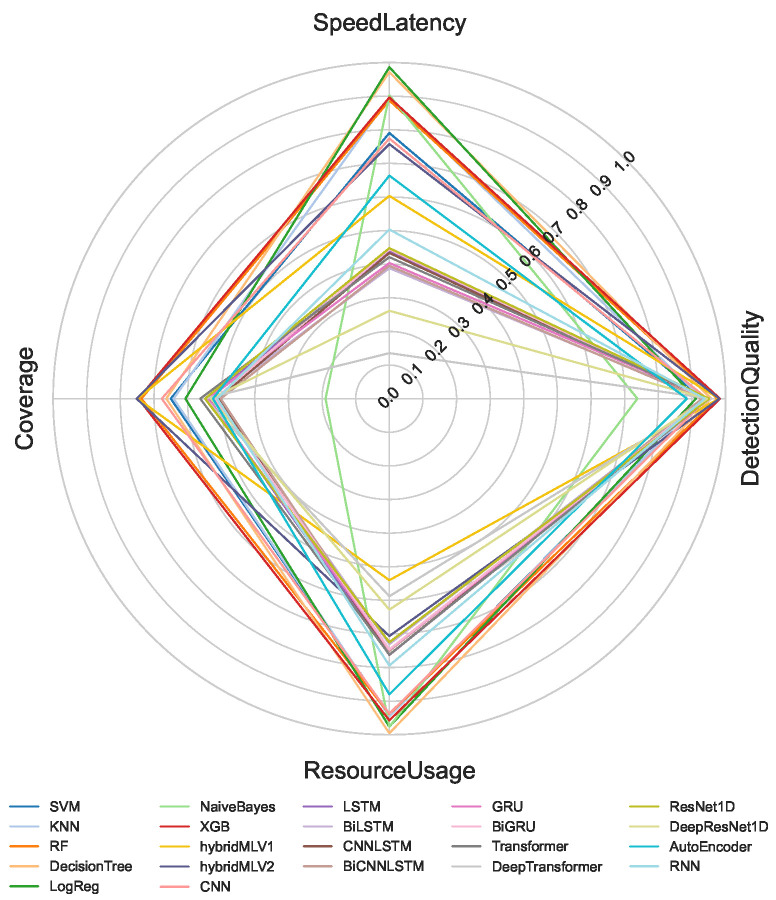
8-class classification.

**Figure 7:**
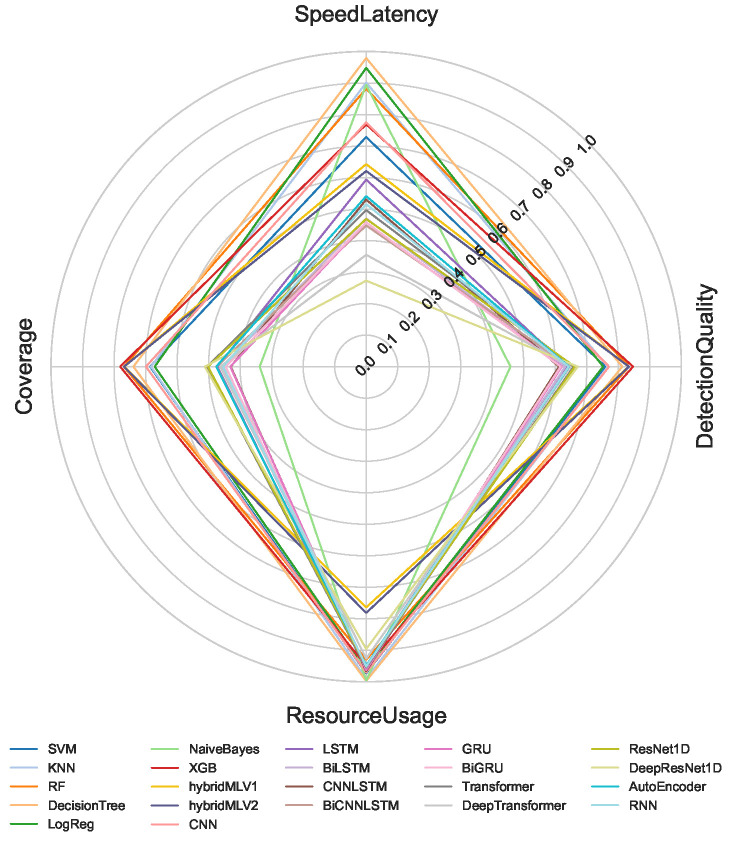
Aggregated performance metrics across models for 50-class classification.

The proposed multi-criteria performance evaluation framework is applied to all NeuroSense and RuleSense components across three detection layers in the DeepSense architecture. For each classification task including binary, 8-class, and 50-class, the corresponding metrics are computed and aggregated to support a unified comparison of heterogeneous detection models. Figure 5, Figure 6 and Figure 7 visualize these aggregated metrics via radar plots, providing a multi-dimensional perspective of model behavior.

These scores are derived using the VIKOR Scoring method (Section 4), capturing trade-offs across conflicting objectives such as accuracy, latency, and computational cost. The radar plots reveal that no single model consistently dominates across all performance dimensions, underscoring the challenge of model selection in constrained IIoT settings.

To manage the complexity of these multi-criteria trade-offs, manual model selection is impractical. Instead, we formulate a multi-objective optimization problem to identify Pareto-optimal model ensembles, as described in Section 4.8. The performance metrics used in the optimization process are normalized to a common scale, enabling fair cross-model comparison.

Table 1 summarizes the normalized scores for all models across the four evaluation criteria: detection quality, resource usage, speed latency, and coverage. Note that RuleSense is evaluated exclusively in the binary classification task at the edge-level detection layer due to its rule-based structure. To improve reliability of the comparison, all values are reported as mean ± standard deviation over repeated executions, allowing confidence intervals to reflect score stability under independent initialization conditions. The observed variance remains limited for most models, indicating that the aggregated rankings are statistically stable and not driven by isolated experimental fluctuations.

A quantitative inspection of Table 1 confirms that no single model achieves dominant performance across all criteria simultaneously. Several architectures exhibit strong specialization but weak overall balance. For example, BiCNNLSTM and GRU achieve comparatively high detection quality, particularly in low- and medium-complexity classification scenarios, yet their lower resource efficiency, slower inference behavior, and weaker coverage reduce their suitability under practical deployment constraints. Similarly, certain lightweight models preserve favorable resource usage but experience substantial degradation in detection quality as class granularity increases.

In contrast, a smaller group of models demonstrates consistently balanced performance across all dimensions. RuleSense, XGBoost, RandomForest, Decision Tree, and CNN maintain comparatively strong aggregate behavior by preserving high detection quality while avoiding excessive penalties in latency, computational demand, or coverage. Among these, tree-based methods such as XGBoost, RandomForest, and Decision Tree provide particularly stable cross-scenario behavior because their detection quality remains high while computational efficiency stays favorable under increasing class complexity. CNN also maintains competitive balance, especially when moving from binary to multiclass settings, indicating stronger feature generalization under moderate computational cost.

RuleSense achieves the strongest binary edge-layer balance because its rule-driven inference produces simultaneously high detection quality and minimal operational uncertainty under the constrained two-class setting. However, because of its rule dependency, this advantage is restricted to binary deployment and does not directly generalize to higher-class scenarios. Overall, the results indicate that the proposed evaluation framework is effective precisely because superiority cannot be inferred from a single metric alone; models that appear strong under one criterion may become suboptimal when evaluated jointly across operational constraints.

### 5.2. Optimization-Based Ensemble Selection Results

To identify model combinations that balance detection effectiveness with system-level constraints, we apply NSGA-II-based multi-objective optimization (Section 4.8) with four objectives: detection quality, resource usage, speed latency, and coverage. The algorithm returns sets of Pareto-optimal ensembles for each detection layer, reflecting the inherent trade-offs in performance dimensions. Table 2 lists the top-selected ensembles for binary, 8-class, and 50-class classification tasks.

**Figure 8:**
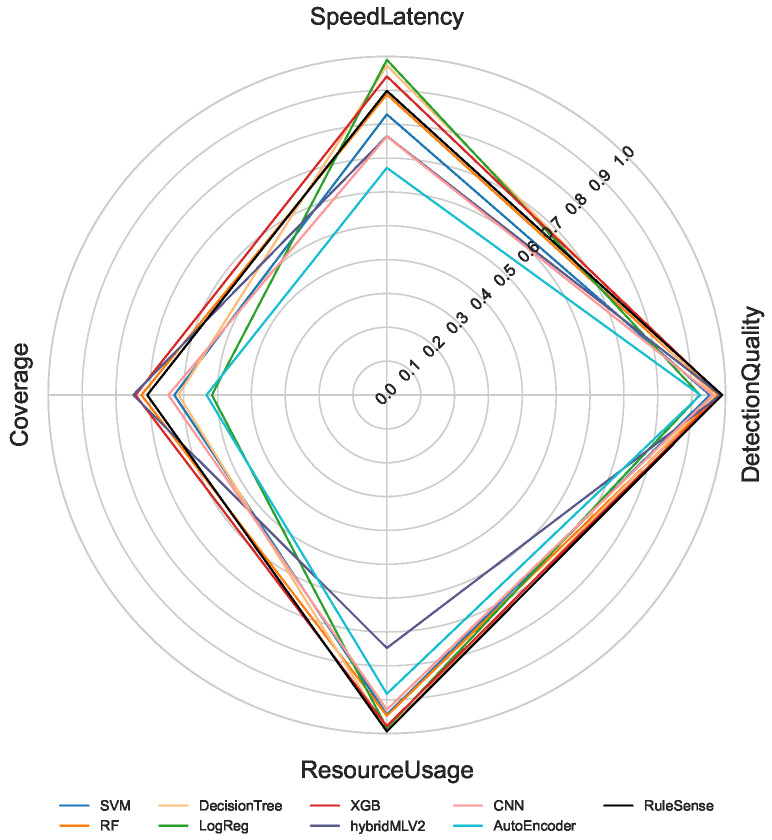
Selected Ensembles (Binary).

Each detection layer employs a layer-specific weighting scheme during optimization. For Layer 1 (edge-level binary detection), the highest priority is assigned to detection quality, followed by coverage, speed latency, and resource usage. RuleSense is consistently selected in all top-ranked Layer 1 ensembles, validating its suitability as an edge-resident detector. Figure 8 visualizes the aggregated performance metrics for the top Layer 1 ensemble candidates.

**Figure 9:**
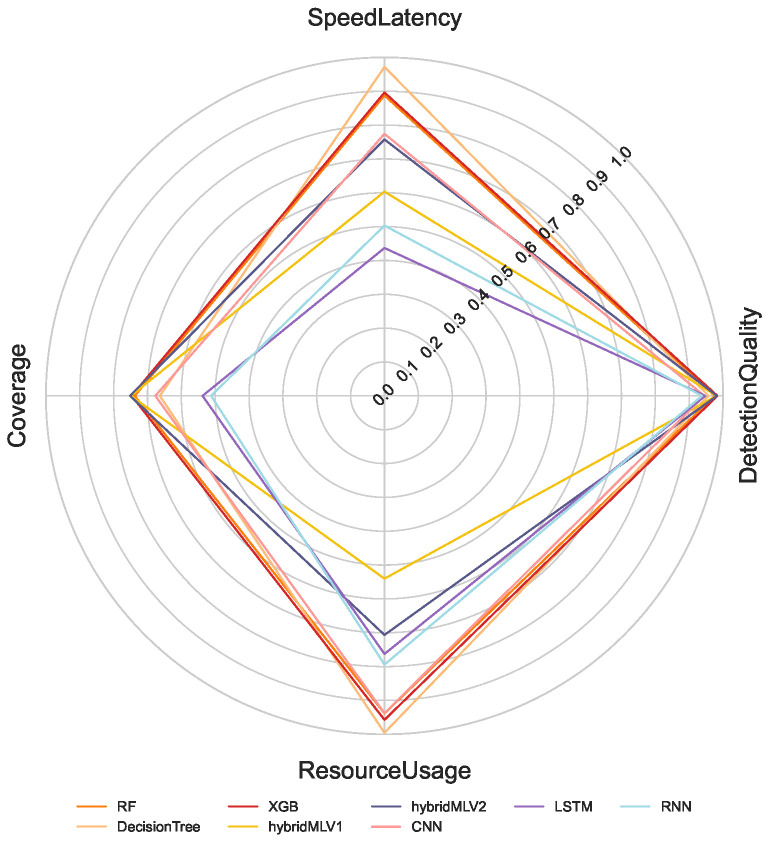
Selected Ensembles (8-Class).

**Figure 10:**
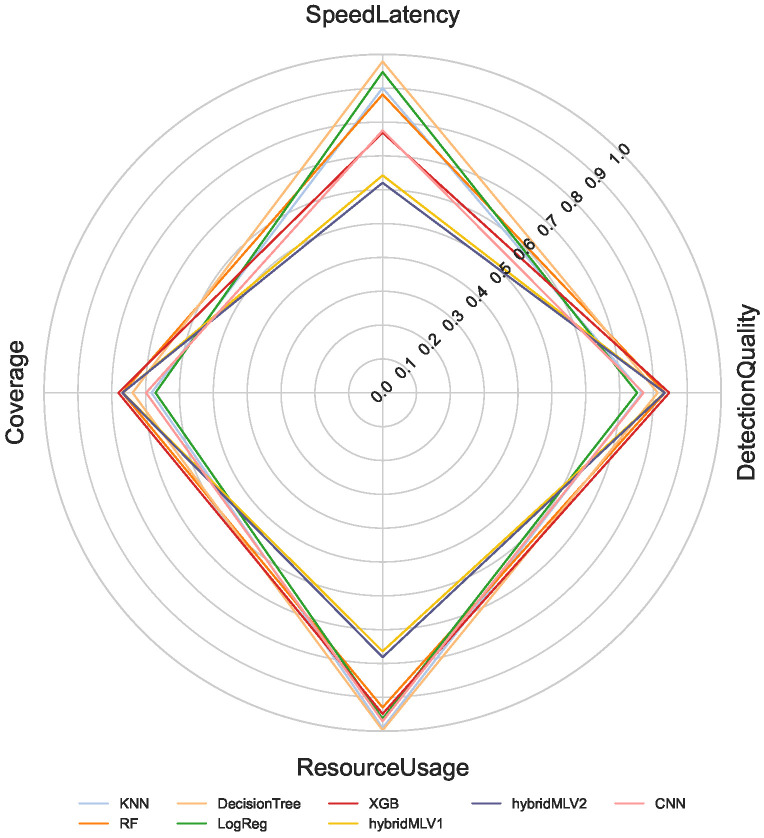
Selected Ensembles (50-Class).

For Layer 2 (cloud-based 8-class classification), the optimization maintains emphasis on detection quality and coverage but allows greater flexibility in computational resource usage, given the cloud deployment context. In Layer 3, which handles 50-class classification task, the prioritization shifts further toward detection quality and coverage, with less emphasis on latency and resource efficiency. This reflects the focus on comprehensive attack characterization after initial detection. The selected ensemble performance profiles for these multi-class tasks are presented in Figure 9 and Figure 10.

To ensure robustness, the ensemble selection procedure was executed 100 times per layer. Final selections correspond to the top three ensembles most frequently identified across all runs, ensuring stable and consistent results despite the stochastic nature of evolutionary optimization.

### 5.3. Sensitivity Analysis of Metric Weighting and Ranking Stability

To assess the robustness of the multi-criteria ranking framework, a sensitivity analysis was conducted by varying the relative importance assigned to the four evaluation dimensions: detection quality, resource usage, speed latency, and coverage. Four alternative weight settings were considered to reflect different deployment priorities. W1 corresponds to the baseline configuration used throughout the main analysis with weights (0.40,0.20,0.20,0.20), W2 applies equal importance to all criteria using (0.25,0.25,0.25,0.25), W3 emphasizes detection quality to represent security-critical deployment conditions using (0.55,0.15,0.15,0.15), and W4 increases the influence of efficiency-related criteria to reflect resource-constrained edge environments using (0.20,0.30,0.30,0.20). In all configurations, the weight order follows detection quality, resource usage, speed latency, and coverage. For each setting, model rankings were recomputed using TOPSIS, VIKOR, and Augmented Chebyshev scoring, and the top three models were compared across the 2-class, 8-class, and 50-class classification scenarios. The resulting rankings are summarized in Table 3.

Across all classification scenarios, XGBoost and Random Forest remain the most consistently top-ranked models under all weighting configurations and aggregation methods, indicating strong ranking stability and balanced multi-criteria performance. RuleSense dominates binary detection when detection quality is prioritized, whereas DecisionTree becomes highly competitive under efficiency-focused settings because of its superior resource usage and latency scores. In the 8-class and 50-class scenarios, hybridML variants appear among the top-ranked candidates when detection quality receives higher emphasis, although their rankings decline when efficiency-related criteria are prioritized. Overall, while minor ordering changes occur across weighting schemes, the same core model family repeatedly remains within the top-ranked set, supporting the robustness of the proposed multi-criteria evaluation framework. The limited variation in top-ranked candidates across these settings indicates that the comparative conclusions are not strongly dependent on a single weighting assumption.

### 5.4. Adaptive Scalable Ensemble Evaluation Results

This section presents an experimental evaluation of the proposed adaptive and scalable ensemble framework under dynamic IIoT operating conditions. The analysis focuses on assessing the framework’s ability to sustain high detection accuracy, robustness, and operational efficiency in the presence of evolving traffic patterns, changing device behavior, and increasing classification complexity. By integrating edge-level rule-based screening with cloud-based ensemble learning and adaptive feedback, the evaluation examines how coordinated multi-layer detection and continuous model refinement contribute to reliable and resilient intrusion detection in real-world IIoT deployments.

#### Baseline Ensemble Performance

**Figure 11:**
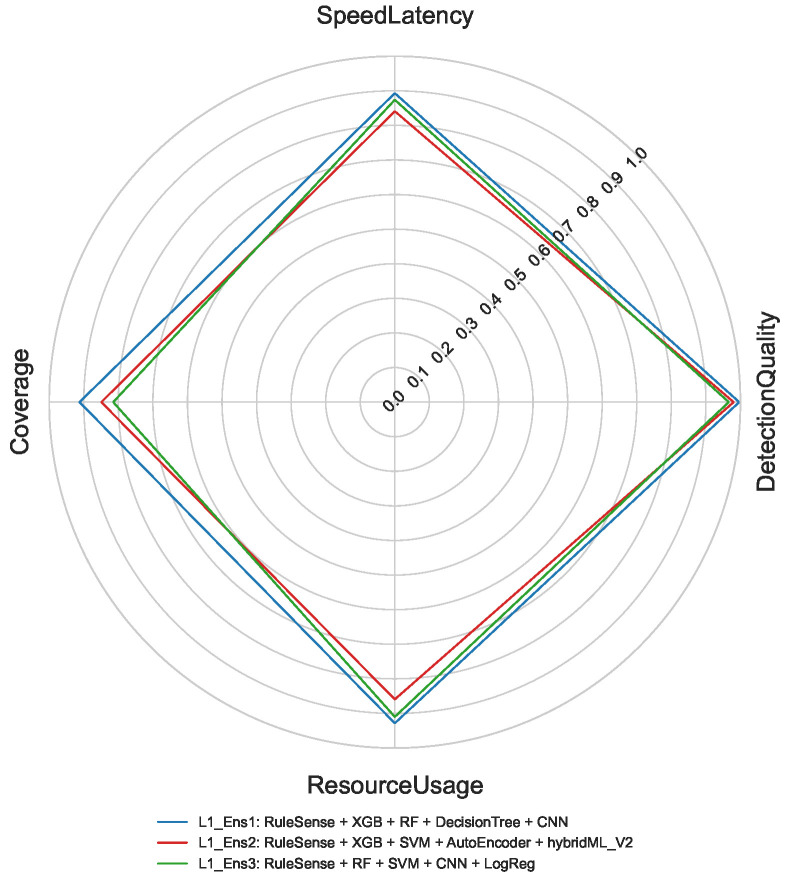
Ensemble Performance Metrics Across Best Selected Ensembles for Binary Classification.

**Figure 12:**
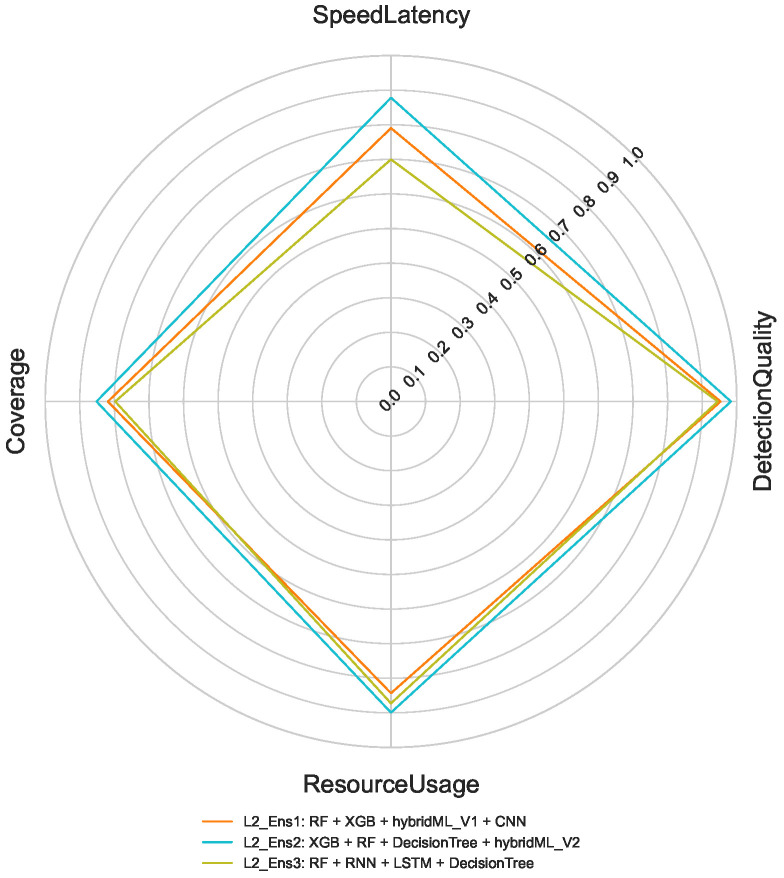
Ensemble Performance Metrics Across Best Selected Ensembles for 8-Class Classification.

**Figure 13:**
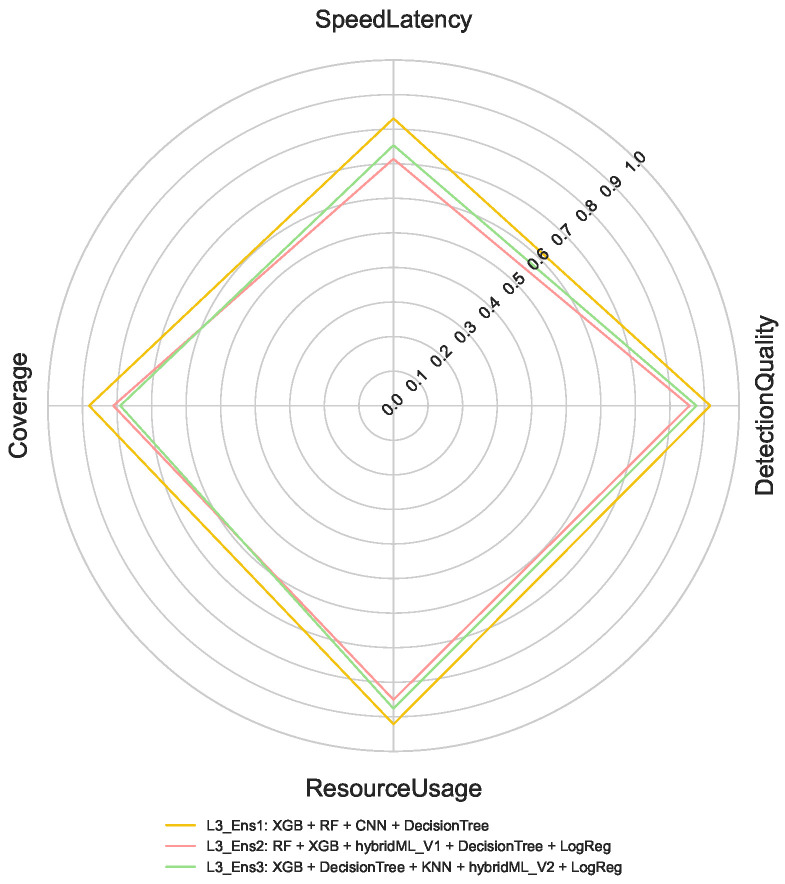
Ensemble Performance Metrics Across Best Selected Ensembles for 50-Class Classification.

This subsection reports the baseline performance of the proposed adaptive ensemble framework using the ensemble configurations summarized in Table 2. A total of nine experiments were conducted, covering three ensemble configurations for each classification scenario, namely binary intrusion detection, 8-class attack category classification, and 50-class attack type classification. For each scenario, ensembles were selected via the optimization procedure and evaluated across all detection layers. The resulting performance profiles are illustrated in Figure 11, Figure 12 and Figure 13.

Ensemble inference follows a weighted aggregation of *M* constituent models,

$$\hat{y}=\arg\max_{c}\sum_{i=1}^{M}w_{i}\;p_{i}\left(y=c|x\right),$$

where $p_{i}\left(y|x\right)$ denotes the posterior output of the *i*-th model and $w_{i}$ represents its optimized contribution weight. Performance is evaluated using DetectionQuality, Coverage, SpeedLatency, and ResourceUsage, reflecting both detection effectiveness and operational efficiency.

For binary classification, Figure 11 shows that L1_Ens1 achieves the best overall performance. Although ensemble execution introduces moderate latency and resource overhead due to parallel model execution and fusion, substantial gains are observed in DetectionQuality and Coverage compared to single-model baselines.

In the 8-class attack category scenario, L2_Ens2 yields the highest performance across all metrics, as illustrated in Figure 12. The results confirm that ensemble diversity significantly improves multi-class discrimination, with only marginal increases in computational cost.

For the 50-class attack type task, Figure 13 indicates that L3_Ens1 delivers the strongest performance. Despite increased classification granularity, the ensemble maintains high DetectionQuality and Coverage, demonstrating scalability and robustness under high-resolution classification demands.

### 5.5. Comparative Performance Against Recent IIoT Detection Frameworks

Table 4 presents a direct comparison of DeepSense against both internal single-model baselines and representative recent IIoT intrusion detection frameworks. Across all tasks, the ensemble consistently outperforms both the best individual model (Max) and the average baseline (Mean) in Accuracy, Precision, Recall, F1-score, and Matthews Correlation Coefficient (MCC), confirming that the fusion strategy improves robustness and reduces model-specific bias. These gains become increasingly pronounced as task complexity increases, with particularly strong improvements observed under the 8-class and 50-class settings, where ensemble learning substantially improves generalization under finer attack granularity.

For binary detection, DeepSense also achieves stronger overall performance than recent representative approaches, particularly in F1-score and MCC, while maintaining highly competitive recall. The comparison further shows that most recent IIoT intrusion detection studies primarily focus on binary anomaly detection and do not report equivalent multi-class evaluations, making direct external comparison beyond binary settings limited. For the multi-class tasks, DeepSense therefore remains compared against internal baselines, where the ensemble improves accuracy by +0.60 %p and F1-score by +0.54 %p in the 8-class scenario, and by +9.04 %p and +9.06 %p, respectively, in the 50-class scenario. This confirms that the benefit of fusion becomes more pronounced as attack discrimination grows more challenging, which is particularly important in practical IIoT environments where identifying attack type is critical for response and mitigation.

#### 5.5.1. Scalability and Robustness Under Deployment Constraints

This subsection evaluates the scalability and robustness of the proposed adaptive ensemble framework under constrained training conditions that reflect realistic IIoT deployment scenarios. In practical settings, intrusion detection systems are frequently initialized with data from a limited number of devices and a restricted subset of known attack types, while being required to generalize to unseen devices and emerging threats.

**Figure 14:**
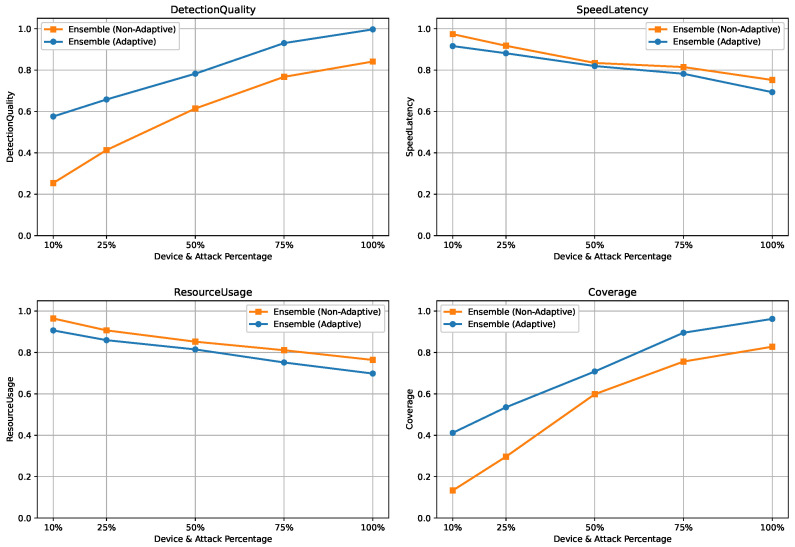
Scalability results for binary classification under varying device and attack availability.

**Figure 15:**
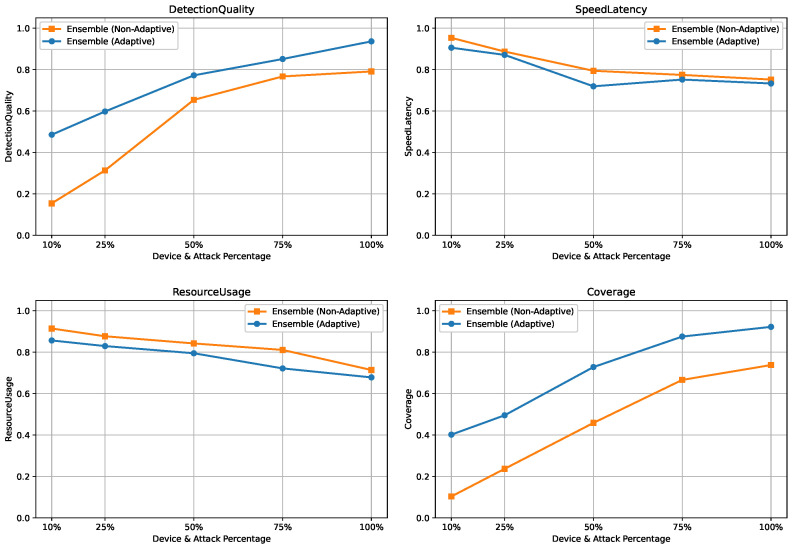
Scalability results for 8-class classification under varying device and attack availability.

**Figure 16:**
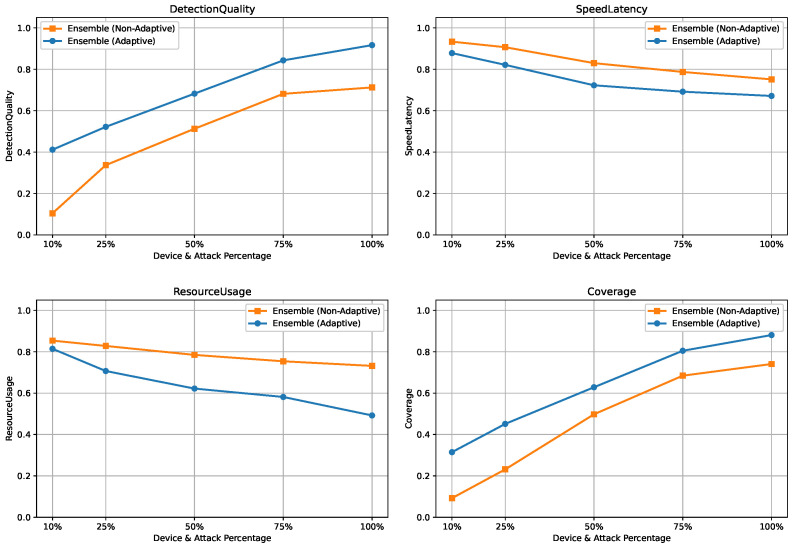
Scalability results for 50-class classification under varying device and attack availability.

To emulate this scenario, the dataset, comprising approximately 40 devices and 50 attack types, was jointly partitioned along both device and attack dimensions. For each experiment, only {10%,25%,50%,75%,100%} of devices and attack types were used for training and profiling, while the remaining unseen devices and attacks were reserved exclusively for evaluation. For each constraint level, models and rule-based profiles were trained on the selected subset and evaluated on unseen data across three tasks, namely binary detection, 8-class attack category classification, and 50-class attack type classification. The results are summarized in Figure 14, Figure 15 and Figure 16.

Two configurations are compared: a static ensemble with fixed models, and the proposed adaptive ensemble with drift monitoring and retraining. Let $P_{t}\left(X,Y\right)$ denote the training distribution and $P_{t+\Delta }\left(X,Y\right)$ the deployment distribution. Constrained training increases the divergence $P_{t}\neq P_{t+\Delta }$, leading to performance degradation in static ensembles. The adaptive framework mitigates this effect by periodically minimizing

$$\min_{\theta }\;E_{\left(X,Y\right)∼P_{t+\Delta }}\left[L(f_{\theta }\left(X\right),Y)\right],$$

using samples accumulated through drift-triggered feedback.

#### 5.5.2. Binary Classification Under Device and Attack Constraints

Figure 14 shows that under severe constraints, static ensembles suffer from low DetectionQuality and Coverage due to poor generalization to unseen devices and attacks. Enabling adaptive learning significantly improves detection performance by updating rules and models in response to drift, with only modest increases in SpeedLatency and ResourceUsage.

#### 5.5.3. Attack Category Classification Under Device and Attack Constraints

As illustrated in Figure 15, performance degradation under limited training coverage is more pronounced for the 8-class task due to increased class diversity and the absence of rule-based filtering. Nevertheless, adaptive learning consistently preserves higher DetectionQuality and Coverage by realigning the ensemble with evolving device and attack distributions.

#### 5.5.4. Attack Type Classification Under Device and Attack Constraints

Figure 16 presents results for the 50-class attack type task, which represents the most challenging scenario. Static ensembles exhibit sharp declines in DetectionQuality and Coverage under restricted training data. In contrast, the adaptive ensemble maintains substantially higher performance by retraining on drift-indicative samples, demonstrating robustness and scalability despite increased latency and resource demands.

Overall, these results confirm that the proposed adaptive ensemble effectively mitigates performance degradation caused by limited device and attack coverage, ensuring scalable and robust detection under realistic IIoT deployment constraints.

#### 5.5.5. Cross-Dataset Validation on Public IIoT Benchmarks

**Figure 17:**
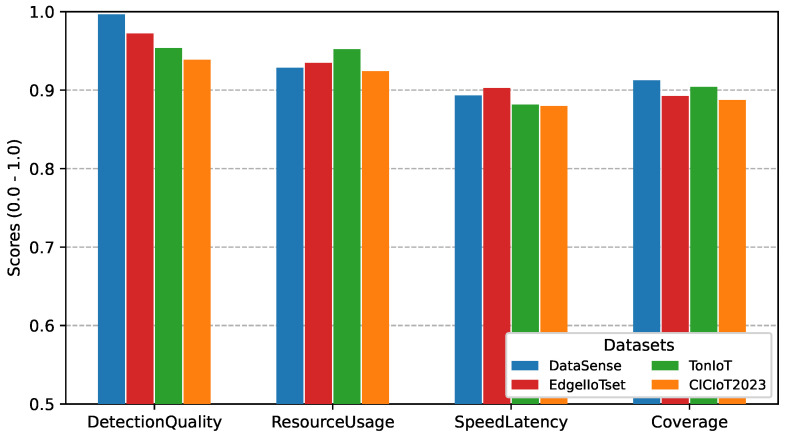
Cross-dataset binary detection performance on EdgeIIoTset, TonIoT, and CICIoT2023 using selected DeepSense ensembles.

**Figure 18:**
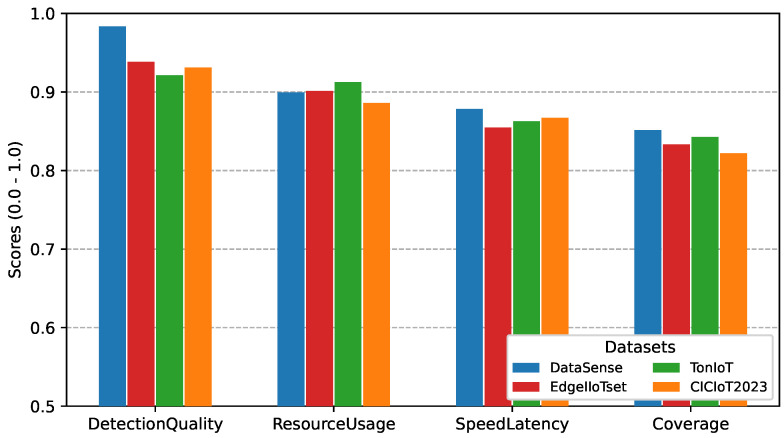
Cross-dataset category classification performance on EdgeIIoTset, TonIoT, and CICIoT2023 using selected DeepSense ensembles.

To further assess generalization beyond the proposed dataset, DeepSense was evaluated on three publicly available IIoT intrusion detection datasets, namely EdgeIIoTset, TonIoT, and CICIoT2023. For consistency, the same evaluation framework and normalized performance criteria were applied to the binary detection layer (L1) and attack-category classification layer (L2). The corresponding results are presented in Figure 17 and Figure 18.

The results show that DeepSense maintains strong performance across all evaluated datasets, with binary detection consistently achieving high DetectionQuality above 0.93 and ResourceUsage remaining above 0.92 in all cases. As expected, L2 classification exhibits a moderate decrease compared with L1 due to increased class complexity, yet DetectionQuality remains above 0.92 across all public datasets. EdgeIIoTset shows performance closest to DataSense, while TonIoT and CICIoT2023 introduce slightly larger reductions, mainly in coverage and latency-related criteria. Overall, the limited variation across datasets indicates that the proposed framework preserves stable behavior under different IIoT traffic characteristics and attack distributions, supporting its generalization capability beyond the original experimental environment.

#### 5.5.6. High-Throughput and Stress-Test Evaluation

To complement the generalization-oriented scalability analysis, we also evaluated the runtime behavior of DeepSense under increasing workload intensity by replaying the evaluation stream at progressively accelerated rates from 1× to 10×, where 1× corresponds to the original observed sample arrival rate of the dataset. For each replay level, throughput, average latency, CPU utilization, memory usage, and false alarm stability were measured while the full adaptive pipeline, including drift monitoring, remained active. Throughput denotes the number of samples fully processed per second, latency represents the average end-to-end decision time per sample, CPU and memory usage capture computational and memory requirements during inference, and false alarm rate reflects the stability of detection decisions by accounting for both false positive and false negative outcomes under increasing load.

**Figure 19:**
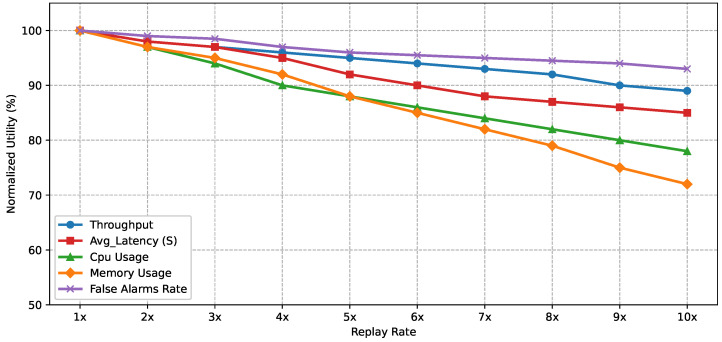
Normalized utility values of throughput, latency, CPU usage, memory usage, and false alarm rate under increasing replay intensity, illustrating operational stability under high-throughput conditions.

Figure 19 shows that throughput remains highly stable, decreasing only from 100% to 89% at 10× replay, while false alarm rate exhibits only a minor reduction from 100% to 93%, indicating that detection reliability is largely preserved under high input intensity. In contrast, latency and resource-related utility values gradually decline, reflecting the expected increase in computational demand. Due to the scalable modular design of DeepSense, API-based model services and detection modules can be scaled up/down under different deployment loads, allowing the framework to maintain stable operational behavior despite increased resource consumption.

### 5.6. Explainability and Decision Transparency

DeepSense incorporates an explainability layer that exposes intermediate decision evidence together with the final detection outcome. At the edge layer, RuleSense produces a binary verdict, confidence score, and feature-level threshold violations for each analyzed sample. Since IIoT devices exhibit distinct operational behavior, normal feature ranges are profiled independently for each device type, allowing threshold violations to be interpreted relative to device-specific operational baselines rather than a global threshold.

To improve decision transparency, confidence scores generated by participating ML/DL models in higher layers are preserved and attached to the same sample, enabling direct comparison between rule-based evidence and model-level confidence. This combined representation helps analysts determine whether a detection is supported by explicit feature violations, cross-model agreement, or conflicting model behavior.

Beyond interpretability, this mechanism supports practical framework refinement by enabling rapid inspection of repeatedly triggered features, threshold adjustment, and device-profile updates without requiring raw packet-level re-analysis. Typical indicators include abnormal port diversity, packet-size statistics, transport flag activity, and inter-packet timing patterns. A representative structured explainability output is provided in Appendix B.

## 6. Conclusions and Future Work

This paper presented DeepSense, a hybrid and adaptive anomaly detection framework for Industrial Internet of Things (IIoT) environments that integrates rule-based and learning-driven detection into a unified, multi-layer architecture. By combining fast edge-level screening through RuleSense with an adaptive ensemble of 22 machine learning and deep learning models in NeuroSense, DeepSense achieves robust detection across multiple classification granularities while remaining suitable for resource-constrained deployments.

Experimental results demonstrate that the proposed adaptive ensemble consistently outperforms isolated detection models across all evaluated scenarios. For binary classification, the ensemble achieved an accuracy of 99.71% and an MCC of 99.41%, exceeding the best individual models. In the 8-class attack category task, the ensemble improved accuracy to 99.12%, while maintaining high precision and recall, indicating enhanced robustness under moderate class imbalance. The benefits of adaptive ensembling become most pronounced in the fine-grained 50-class scenario, where the ensemble improved accuracy from 86.01% (best single model) to 95.05%, and increased MCC from 82.39% to 90.07%, highlighting its effectiveness in capturing subtle and overlapping attack behaviors. These results confirm that adaptive ensemble learning significantly improves generalization, stability, and detection reliability compared to standalone models.

Overall, DeepSense provides a scalable, efficient, and resilient IIoT security solution that balances detection quality, latency, and resource efficiency, making it well aligned with the operational requirements of Industry 4.0 and the resilience-driven objectives of Industry 5.0.

While DeepSense establishes a strong foundation for adaptive IIoT intrusion detection, several promising research directions remain. First, extending the framework toward federated and decentralized learning would enable collaborative detection across distributed edge devices while preserving data privacy and improving scalability. Second, integrating explainability and transparency mechanisms into the ensemble decision process could enhance operator trust and support more effective incident analysis in safety-critical environments. Finally, further investigation into advanced adaptive re-training and re-profiling strategies is needed to better handle long-term concept drift while minimizing computational overhead and resilience against noisy or adversarial inputs. These directions will further strengthen DeepSense’s applicability to real-world industrial deployments.

## Figures and Tables

**Table 1:** Aggregated performance criteria values for all evaluated models across different classification scenarios (2, 8, and 50 classes).

Algorithm	Scenario	Detection Quality	Resource Usage	Speed Latency	Coverage
SVM	2 Class	**0.9539** ± 0.0147	**0.9443** ± 0.0368	**0.8291** ± 0.0523	**0.6278** ± 0.0416
	8 Class	**0.9323** ± 0.0214	**0.9427** ± 0.0485	**0.7907** ± 0.0679	**0.6501** ± 0.0338
	50 Class	**0.7585** ± 0.0386	**0.9731** ± 0.0712	**0.7285** ± 0.0847	**0.6855** ± 0.0569
KNN	2 Class	**0.9528** ± 0.0879	**0.9035** ± 0.0941	**0.8906** ± 0.1248	**0.6116** ± 0.0394
	8 Class	**0.9319** ± 0.0743	**0.9450** ± 0.0616	**0.8964** ± 0.1387	**0.6420** ± 0.0472
	50 Class	**0.7669** ± 0.1118	**0.9909** ± 0.0927	**0.9010** ± 0.0815	**0.6828** ± 0.1221
RandomForest	2 Class	**0.9782** ± 0.0116	**0.9476** ± 0.0394	**0.8879** ± 0.0432	**0.7248** ± 0.0267
	8 Class	**0.9753** ± 0.0138	**0.9381** ± 0.0569	**0.8874** ± 0.0493	**0.7376** ± 0.0315
	50 Class	**0.8339** ± 0.0271	**0.9298** ± 0.0674	**0.8818** ± 0.0586	**0.7689** ± 0.0437
DecisionTree	2 Class	**0.9658** ± 0.0152	**0.9923** ± 0.0286	**0.9737** ± 0.0341	**0.6152** ± 0.0379
	8 Class	**0.9570** ± 0.0197	**0.9957** ± 0.0248	**0.9718** ± 0.0295	**0.6635** ± 0.0413
	50 Class	**0.8127** ± 0.0349	**0.9974** ± 0.0196	**0.9791** ± 0.0257	**0.7387** ± 0.0546
LogReg	2 Class	**0.9246** ± 0.0264	**0.9838** ± 0.0337	**0.9902** ± 0.0246	**0.5167** ± 0.0289
	8 Class	**0.9120** ± 0.0315	**0.9760** ± 0.0418	**0.9865** ± 0.0312	**0.6065** ± 0.0438
	50 Class	**0.7520** ± 0.0374	**0.9634** ± 0.0591	**0.9479** ± 0.0486	**0.6710** ± 0.0528
NaiveBayes	2 Class	**0.8864** ± 0.0547	**0.9716** ± 0.1463	**0.9464** ± 0.0717	**0.3295** ± 0.0686
	8 Class	**0.7380** ± 0.0682	**0.9766** ± 0.1321	**0.9027** ± 0.0978	**0.1898** ± 0.0349
	50 Class	**0.4580** ± 0.1093	**0.9961** ± 0.0418	**0.8941** ± 0.0736	**0.3379** ± 0.0324
XGB	2 Class	**0.9863** ± 0.0119	**0.9783** ± 0.0275	**0.9415** ± 0.0376	**0.7423** ± 0.0258
	8 Class	**0.9833** ± 0.0128	**0.9573** ± 0.0439	**0.8962** ± 0.0475	**0.7464** ± 0.0287
	50 Class	**0.8468** ± 0.0267	**0.9488** ± 0.0613	**0.7690** ± 0.0715	**0.7806** ± 0.0396
hybridML_V1	2 Class	**0.9861** ± 0.0124	**0.6460** ± 0.0792	**0.6000** ± 0.0838	**0.7503** ± 0.0314
	8 Class	**0.9809** ± 0.0143	**0.5402** ± 0.0847	**0.6034** ± 0.0785	**0.7488** ± 0.0279
	50 Class	**0.8327** ± 0.0298	**0.3639** ± 0.0816	**0.3425** ± 0.0893	**0.7676** ± 0.0418
hybridML_V2	2 Class	**0.9856** ± 0.0131	**0.7474** ± 0.0684	**0.7648** ± 0.0547	**0.7492** ± 0.0261
	8 Class	**0.9819** ± 0.0149	**0.7064** ± 0.0728	**0.7576** ± 0.0596	**0.7516** ± 0.0295
	50 Class	**0.8337** ± 0.0284	**0.5315** ± 0.0773	**0.6206** ± 0.0739	**0.7686** ± 0.0384
CNN	2 Class	**0.9620** ± 0.0168	**0.9296** ± 0.0427	**0.7641** ± 0.0574	**0.6454** ± 0.0346
	8 Class	**0.9449** ± 0.0216	**0.9376** ± 0.0493	**0.7742** ± 0.0618	**0.6768** ± 0.0415
	50 Class	**0.7706** ± 0.0337	**0.9705** ± 0.0678	**0.7754** ± 0.0731	**0.6987** ± 0.0529
LSTM	2 Class	**0.9698** ± 0.0149	**0.7602** ± 0.0734	**0.4303** ± 0.0817	**0.3630** ± 0.0264
	8 Class	**0.9475** ± 0.0198	**0.7626** ± 0.0689	**0.4369** ± 0.0763	**0.5374** ± 0.0387
	50 Class	**0.6376** ± 0.0351	**0.9623** ± 0.0596	**0.5934** ± 0.0694	**0.4718** ± 0.0458
BiLSTM	2 Class	**0.9742** ± 0.0437	**0.7229** ± 0.0776	**0.3791** ± 0.0849	**0.3687** ± 0.1198
	8 Class	**0.9380** ± 0.0524	**0.7305** ± 0.0713	**0.3884** ± 0.0796	**0.5201** ± 0.0371
	50 Class	**0.6318** ± 0.1268	**0.9509** ± 0.0828	**0.4699** ± 0.0711	**0.4578** ± 0.0633
CNNLSTM	2 Class	**0.9760** ± 0.0529	**0.7623** ± 0.0697	**0.4326** ± 0.0784	**0.3759** ± 0.0612
	8 Class	**0.9355** ± 0.1137	**0.7621** ± 0.0665	**0.4336** ± 0.0938	**0.5080** ± 0.0596
	50 Class	**0.6118** ± 0.0784	**0.9664** ± 0.0867	**0.5301** ± 0.1471	**0.4297** ± 0.0974
BiCNNLSTM	2 Class	**0.9837** ± 0.1118	**0.7364** ± 0.1729	**0.3990** ± 0.1815	**0.3953** ± 0.0887
	8 Class	**0.9303** ± 0.1248	**0.7506** ± 0.1687	**0.3954** ± 0.0774	**0.5017** ± 0.0665
	50 Class	**0.6463** ± 0.1042	**0.9564** ± 0.0814	**0.4479** ± 0.0993	**0.4746** ± 0.0721
GRU	2 Class	**0.9675** ± 0.0354	**0.7710** ± 0.0673	**0.4419** ± 0.0746	**0.3518** ± 0.0249
	8 Class	**0.9521** ± 0.0187	**0.7470** ± 0.0718	**0.4036** ± 0.0761	**0.5392** ± 0.0388
	50 Class	**0.6202** ± 0.0376	**0.9624** ± 0.0583	**0.4577** ± 0.0688	**0.4311** ± 0.0446
BiGRU	2 Class	**0.9629** ± 0.0363	**0.7569** ± 0.0716	**0.4140** ± 0.0783	**0.2738** ± 0.0217
	8 Class	**0.9409** ± 0.0521	**0.7463** ± 0.0694	**0.4445** ± 0.0739	**0.5176** ± 0.0374
	50 Class	**0.6171** ± 0.1079	**0.9555** ± 0.0808	**0.4579** ± 0.0972	**0.4457** ± 0.0439
Transformer	2 Class	**0.9404** ± 0.0218	**0.7695** ± 0.0667	**0.4618** ± 0.0725	**0.1813** ± 0.0142
	8 Class	**0.9495** ± 0.0184	**0.7418** ± 0.0703	**0.3912** ± 0.0791	**0.5324** ± 0.0391
	50 Class	**0.6579** ± 0.0346	**0.9546** ± 0.0617	**0.4965** ± 0.0684	**0.5043** ± 0.0418
DeepTransformer	2 Class	**0.9703** ± 0.0141	**0.5815** ± 0.0824	**0.1566** ± 0.0879	**0.3359** ± 0.0248
	8 Class	**0.9564** ± 0.0176	**0.5880** ± 0.0791	**0.1355** ± 0.0894	**0.5481** ± 0.0386
	50 Class	**0.6481** ± 0.0358	**0.9264** ± 0.0643	**0.3549** ± 0.0816	**0.4629** ± 0.0445
ResNet1D	2 Class	**0.9750** ± 0.0133	**0.7375** ± 0.0731	**0.3999** ± 0.0776	**0.3567** ± 0.0273
	8 Class	**0.9513** ± 0.0189	**0.7249** ± 0.0748	**0.3879** ± 0.0802	**0.5481** ± 0.0368
	50 Class	**0.6618** ± 0.0334	**0.9495** ± 0.0621	**0.4686** ± 0.0697	**0.5020** ± 0.0427
DeepResNet1D	2 Class	**0.9714** ± 0.0146	**0.6583** ± 0.0789	**0.2885** ± 0.0843	**0.3509** ± 0.0268
	8 Class	**0.9451** ± 0.0207	**0.6279** ± 0.0814	**0.2616** ± 0.0876	**0.5304** ± 0.0382
	50 Class	**0.6712** ± 0.0331	**0.8958** ± 0.0662	**0.2728** ± 0.0837	**0.5122** ± 0.0434
AutoEncoder	2 Class	**0.9261** ± 0.0249	**0.8828** ± 0.0518	**0.6712** ± 0.0637	**0.5327** ± 0.0375
	8 Class	**0.8851** ± 0.0314	**0.8798** ± 0.0546	**0.6638** ± 0.0661	**0.5244** ± 0.0392
	50 Class	**0.6434** ± 0.0357	**0.9485** ± 0.0616	**0.5410** ± 0.0713	**0.4758** ± 0.0448
RNN	2 Class	**0.9729** ± 0.0139	**0.7860** ± 0.0664	**0.4490** ± 0.0731	**0.3569** ± 0.0259
	8 Class	**0.9378** ± 0.0228	**0.7941** ± 0.0638	**0.5033** ± 0.0695	**0.5121** ± 0.0377
	50 Class	**0.6421** ± 0.0363	**0.9514** ± 0.0594	**0.5158** ± 0.0679	**0.4539** ± 0.0436
RuleSense	2 Class	**0.9904** ± 0.0102	**0.9937** ± 0.0126	**0.8685** ± 0.0219	**0.6578** ± 0.0184

“Classes” indicates the number of target classes in each scenario; ± indicates standard deviation over five repeated runs. “Bold Values”: Base performance of the models, ±: standard deviation of model performance over repeated executions.

**Table 2:** Top-ranked ensemble selections identified by the optimization-based selection for different detection tasks.

Ens ID	Task	Selected Ensemble Components
L1_Ens1	2-Class	RuleSense + XGBoost + Random Forest + DecisionTree + CNN
L1_Ens2	2-Class	RuleSense + XGBoost + SVM + AutoEncoder + hybridML_V2
L1_Ens3	2-Class	RuleSense + Random Forest + SVM + CNN + LogReg
L2_Ens1	8-Class	Random Forest + XGBoost + hybridML_V1 + CNN
L2_Ens2	8-Class	XGBoost + Random Forest + DecisionTree + hybridML_V2
L2_Ens3	8-Class	Random Forest + RNN + LSTM + DecisionTree
L3_Ens1	50-Class	XGBoost + Random Forest + CNN + DecisionTree
L3_Ens2	50-Class	Random Forest + XGBoost + hybridML_V1 + DecisionTree + LogReg
L3_Ens3	50-Class	XGBoost + DecisionTree + KNN + hybridML_V2 + LogReg

Ens ID: Ensemble ID used in subsequent experiments.

**Table 3:** Sensitivity analysis of top-ranked models under alternative weight and scoring configurations.

Task	Weight Settings	TOPSIS Top 3	VIKOR Top 3	Chebyshev Top 3
2-Class	W1	XGB, RF, RuleSense	RuleSense, XGB, RF	XGB, RF, hybridML_V2
	W2	XGB, RF, hybridML_V2	XGB, RF, RuleSense	XGB, hybridML_V2, RF
	W3	RuleSense, XGB, hybridML_V1	RuleSense, XGB, hybridML_V1	RuleSense, XGB, BiCNNLSTM
	W4	XGB, DecisionTree, RF	DecisionTree, XGB, RF	DecisionTree, XGB, RF
8-Class	W1	XGB, RF, hybridML_V2	XGB, RF, DecisionTree	XGB, RF, hybridML_V2
	W2	XGB, RF, DecisionTree	XGB, DecisionTree, RF	XGB, RF, hybridML_V2
	W3	XGB, hybridML_V2, hybridML_V1	XGB, hybridML_V2, RF	XGB, hybridML_V2, RF
	W4	DecisionTree, XGB, RF	DecisionTree, XGB, RF	DecisionTree, XGB, RF
50-Class	W1	XGB, RF, DecisionTree	XGB, RF, CNN	RF, XGB, DecisionTree
	W2	RF, XGB, DecisionTree	XGB, RF, DecisionTree	RF, XGB, DecisionTree
	W3	XGB, RF, hybridML_V2	XGB, RF, hybridML_V1	XGB, RF, hybridML_V2
	W4	DecisionTree, RF, KNN	DecisionTree, RF, XGB	DecisionTree, RF, KNN

W1: baseline weights; W2: equal weights; W3: detection-quality emphasized; and W4: efficiency emphasized.

**Table 4:** Performance comparison of DeepSense against internal baselines and representative recent IIoT intrusion detection frameworks.

			Metrics					
Task	Work	Approach	Acc	Prec	Rec	F1	MCC	%p Improvement
**Binary** **(L1)**	**DeepSense**	**Ensemble (L1_Ens1)**	**99.71**	**99.65**	99.69	**99.67**	**99.41**	–
	DeepSense	Base Models (Max)	99.28	99.13	99.05	99.09	98.49	–
	DeepSense	Base Models (Mean)	97.27	97.37	97.27	97.28	94.58	–
	Logeswari et al. [38]	IA-IDS	98.67	98.60	98.45	98.51	NR	**+1.16 (F1)**
	Al Rawajbeh et al. [17]	Trustworthy AI	96.40	96.10	95.70	95.90	NR	**+3.31 (Acc)**
	Mohy-Eddine et al. [20]	RF-PCCIF	99.30	85.18	99.87	91.94	NR	**+7.73 (F1)**
	Yang and Shami [30]	MSANA	98.88	98.88	**99.94**	99.41	NR	**+0.83 (Acc)**
**8-Class** **(L2)**	**DeepSense**	**Ensemble (L2_Ens2)**	**99.12**	**98.85**	**99.03**	**98.94**	**98.19**	–
	DeepSense	Base Models (Max)	98.52	98.53	98.49	98.40	97.97	–
	DeepSense	Base Models (Mean)	94.36	94.72	94.36	94.42	92.27	–
**50-Class** **(L3)**	**DeepSense**	**Ensemble (L3_Ens1)**	**95.05**	**94.62**	**94.94**	**94.78**	**90.07**	–
	DeepSense	Base Models (Max)	86.01	85.55	86.01	85.72	82.39	–
	DeepSense	Base Models (Mean)	72.43	73.21	72.41	71.65	65.43	–

%p indicates percentage-point improvement of DeepSense over the compared recent work. NR indicates metrics not reported.

**Figure A1:**
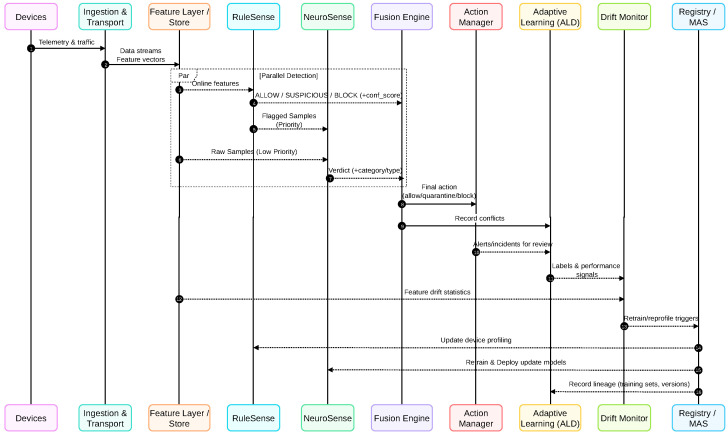
Sequence diagram of end-to-end Deepsense workflow.

## Data Availability

The DataSense dataset used in this study is publicly available through the CIC Dataset portal (https://www.unb.ca/cic/datasets/iiot-dataset-2025.html (accessed on 19 April 2026)). Detailed information regarding the physical testbed, implementation environment, data collection process, and attack generation methodology is provided in [34].

## Appendix A. DeepSense Operational Workflow

Figure A1 presents the end-to-end operational workflow of DeepSense, illustrating how sensing data, detection modules, fusion logic, and adaptation mechanisms interact during real-time operation. Industrial devices continuously generate telemetry and traffic streams, which are collected by the ingestion layer and transformed into normalized feature vectors by the feature layer for online analysis.

Detection then starts in parallel. RuleSense performs immediate profile-based evaluation and assigns an initial verdict (allow, suspicious, or block) together with a confidence score. Suspicious samples are prioritized and forwarded to NeuroSense, where trained models perform deeper analysis to confirm anomalies and determine the corresponding attack category or attack type.

Outputs from RuleSense and NeuroSense are combined by the fusion engine, which resolves agreement or disagreement between both detectors and generates the final decision. The action manager then produces operational commands such as allowing traffic, quarantining devices, or blocking activity. Detection conflicts are recorded and later used as difficult cases for adaptive refinement.

Operational feedback is continuously collected through analyst-reviewed alerts, performance signals, and feature drift statistics. When behavioral drift or repeated conflicts are detected, retraining or re-profiling is triggered through the registry layer, allowing RuleSense profiles and NeuroSense models to be updated while preserving model lineage and deployment history.

This closed-loop design enables DeepSense to combine immediate edge-level detection, deeper centralized inspection, and continuous adaptation while maintaining explainability and practical deployment suitability.

### Appendix A.1. DataSense: Data Pipeline and Experimental Testbed

DataSense constitutes the data acquisition, processing, and experimentation backbone of the DeepSense framework, enabling realistic evaluation of anomaly and intrusion detection in Industrial IoT environments. The DataSense testbed comprises 40 heterogeneous IoT and IIoT devices, including industrial sensors, actuators, controllers, embedded systems, and commonly deployed connected devices, interconnected through realistic industrial networking components. Using this infrastructure, DataSense captures synchronized sensor-level and network-level data streams that reflect real operational behaviors and interactions across heterogeneous industrial setups.

The dataset was generated through the controlled execution of 50 realistic cyberattacks spanning seven major attack categories, namely Reconnaissance, Denial of Service (DoS), Distributed Denial of Service (DDoS), Web Exploitation, Man-in-the-Middle, Brute force, and Malware, resulting in a balanced mix of benign and malicious traffic representative of real-world conditions. In addition, DataSense incorporates a feature selection methodology aimed at improving detection effectiveness while minimizing computational overhead, supporting deployment in resource-constrained environments.

Since the detailed design of the testbed, data generation process, feature engineering pipeline, and extensive validation experiments have been presented in our previous work [34], this paper focuses on the integration role of DataSense within the overall DeepSense architecture. Interested readers are referred to the DataSense publication [34] for comprehensive technical and experimental details.

### Appendix A.2. RuleSense: Lightweight Rule-Based Detection Layer

RuleSense constitutes the rule-based detection core of the DeepSense framework and is designed to provide fast, deterministic, and interpretable anomaly detection at the edge of IIoT environments. It operates as the first line of defense by leveraging device-specific behavioral profiles, protocol-aware rules, and statistical thresholds to continuously monitor sensor and network data streams and to identify deviations from expected behavior in real time. By relying on profiling rather than training-based learning, RuleSense enables low-latency detection with minimal computational overhead, making it well suited for deployment on resource-constrained IIoT devices.

Within the DeepSense architecture, RuleSense performs early-stage anomaly screening and coarse attack identification, allowing suspicious or anomalous samples to be selectively forwarded to the learning-driven NeuroSense module for deeper analysis and fine-grained classification. This layered design reduces unnecessary processing, limits false positives, and improves overall system responsiveness while preserving interpretability and operational transparency.

In this paper, RuleSense is described at a high level to clarify its functional role and interaction with other DeepSense components. Detailed descriptions of the profiling mechanisms, rule construction, detection and classification algorithms, and standalone performance evaluation are beyond the scope of this work and will be reported in a dedicated study.

### Appendix A.3. NeuroSense: Learning-Driven Detection and Adaptive Ensemble

NeuroSense represents the machine learning and deep learning intelligence of the DeepSense framework and is responsible for adaptive, data-driven anomaly and intrusion detection in IIoT environments. It incorporates a diverse set of 22 machine learning and deep learning models, spanning Classical Machine Learning, *Shallow Neural Networks*, Deep Convolutional and Recurrent architectures, Hybrid models, and Transformer-based networks, as summarized in Table 1, to capture nonlinear, temporal, and structural patterns in IIoT traffic. These models support multiple detection granularities, including binary classification (benign versus attack), coarse-grained multiclass classification across major attack categories, and fine-grained multiclass classification covering specific attack subtypes.

The individual ML and DL models integrated within NeuroSense were initially implemented and evaluated in our previous work [34], where their standalone detection capabilities and comparative performance were systematically analyzed under different classification scenarios. Building upon these foundational results, the present work extends NeuroSense by organizing the same set of models into an Adaptive Ensemble Learning Framework that operates as a second detection stage alongside RuleSense. In this design, NeuroSense validates suspicious samples flagged at the edge, aggregates complementary model decisions, and provides refined attack category and subtype classification.

Unlike prior work that focused on isolated model performance, this paper emphasizes the adaptive ensemble design, lightweight decision fusion, and optimization-driven model selection under realistic IIoT constraints. The resulting NeuroSense ensemble improves generalization across heterogeneous environments, enhances robustness against evolving and previously unseen attacks, and reduces false positives while maintaining practical efficiency. Detailed ensemble construction, adaptation mechanisms, and empirical evaluation results are presented in the subsequent sections.

## Appendix B. Example Explainability Output

This appendix provides a representative structured explainability output generated by DeepSense for a detected anomalous sample. The output illustrates how device-specific threshold violations identified by RuleSense are combined with confidence scores from higher-layer ML/DL models to provide transparent decision evidence for analyst inspection and framework refinement.

Device: weather-sensor_A12 Predicted verdict: Attack RuleSense confidence: 0.96 Triggered abnormal features:

- network_packet-size_avg = 71.38 (normal threshold: 62.00)
- network_ports_all_count = 2501 (normal threshold: 180)
- network_time-delta_avg = 3.07 × 10^−5^ (normal threshold: 1.20 × 10^−3^ )
- network_tcp-flags-psh_count = 2500 (normal threshold: 320)

ML/DL confidence scores:

- RandomForest: 0.97
- XGBoost: 0.95
- CNN: 0.93

Final ensemble confidence: 0.96

**Example of explained detected sample:**

{

 "device_id": "weather-sensor_A12",

 "sample_id": "SE01Gh87ADE",

 "predicted_label": "Attack",

 "rulesense_confidence": 0.96,

** **

 "triggered_features": {

  "network_packet-size_avg": {

   "observed": 71.38,

   "normal_threshold": 62.00

  },

  "network_ports_all_count": {

   "observed": 2501,

   "normal_threshold": 180

  },

  "network_time-delta_avg": {

   "observed": 3.07e-05,

   "normal_threshold": 1.20e-03

  },

  "network_tcp-flags-psh_count": {

   "observed": 2500,

   "normal_threshold": 320

  }

 },

** **

 "model_confidence": {

  "RandomForest": 0.97,

  "XGBoost": 0.95,

  "CNN": 0.93

 },

 "ensemble_confidence": 0.96

}
